# Perceptual (but not acoustic) features predict singing voice preferences

**DOI:** 10.1038/s41598-024-58924-9

**Published:** 2024-04-18

**Authors:** Camila Bruder, David Poeppel, Pauline Larrouy-Maestri

**Affiliations:** 1 Max Planck Institute for Empirical Aesthetics, Frankfurt am Main, Germany; 2 New York University, New York, NY USA; 3 Ernst Strüngmann Institute for Neuroscience, Frankfurt, Germany; 4 Max Planck-NYU Center for Language, Music, and Emotion (CLaME), New York, USA

**Keywords:** Human behaviour, Perception

## Abstract

Why do we prefer some singers to others? We investigated how much singing voice preferences can be traced back to objective features of the stimuli. To do so, we asked participants to rate short excerpts of singing performances in terms of how much they liked them as well as in terms of 10 perceptual attributes (e.g.: pitch accuracy, tempo, breathiness). We modeled liking ratings based on these perceptual ratings, as well as based on acoustic features and low-level features derived from Music Information Retrieval (MIR). Mean liking ratings for each stimulus were highly correlated between Experiments 1 (online, US-based participants) and 2 (in the lab, German participants), suggesting a role for attributes of the stimuli in grounding average preferences. We show that acoustic and MIR features barely explain any variance in liking ratings; in contrast, perceptual features of the voices achieved around 43% of prediction. Inter-rater agreement in liking and perceptual ratings was low, indicating substantial (and unsurprising) individual differences in participants’ preferences and perception of the stimuli. Our results indicate that singing voice preferences are not grounded in acoustic attributes of the voices per se, but in how these features are perceptually interpreted by listeners.

## Introduction

Singing is ubiquitous across cultures^1,2^ and highly popular^3^, but fundamental questions about our preferences remain unanswered. For instance: why do we like some singing voices more than others? How much are these preferences based on attributes of the singing voice, and how much are they related to listeners’ internal factors?

The extent to which aesthetic appreciation depends on objective properties of a stimulus versus a person’s internal subjective states and evaluations has long been debated. Pioneering empirical work has been done in the visual domain, starting with Fechner’s investigations of average group preferences for rectangles following the golden ratio^4,5^. More recently, some studies have argued for average preferences for certain stimulus features such as curved versus sharp contours^6^, symmetrical patterns^7^, and high contrast images^8^, among others—though variability across people tends to be large^9^. In fact, for certain types of stimuli (e.g., abstract paintings), individual differences are found to be particularly striking, with small interindividual agreement (or highly idiosyncratic taste) across participants^10–12^, supporting the role of individuals’ internal factors and their personal experiences as critical determinants of their preferences.

In the case of music, a broad range of stimulus features such as complexity, tempo, rhythm, tonal and timbral features, among others, have been shown to predict aesthetic responses to musical sounds (see^13^ for a review). Like in the visual domain, musical preferences are assumed best to be understood with an interactionist approach (e.g.:^14,15^), that is, as the result of a complex interplay of numerous multilevel factors, which can be linked to attributes of the music stimuli themselves (i.e., acoustic features), as well as their perception by listeners (i.e., perceptual features) and other cognitive, emotional, evaluative processing on the listener side.

In the case of the human voice, empirical work has focused on preferences for the spoken voice: studies of vocal attractiveness indicate that the height of fundamental frequency (*f*_*o*_) and formant dispersion of a given voice may function as indirect cues of body size, health, and age, with higher *f*_*o*_ and more spread formants generally preferred for women’s voices and lower *f*_*o*_ preferred for men’s voices^16^. A different line of research has used voice morphing to show that composite, averaged voices (which are smoother and have higher signal-to-noise ratio than individual voices) were considered more attractive than most of the individual voices presented to participants^17^; but see^18^.

Particularly for the singing voice, there is evidence that specific acoustic features influence the perception of pitch accuracy, or of singing performances as “correct”^19^ and that the use of such features is affected by the music expertise of the listeners^20,21^—but such research does not answer questions about singing preferences. In fact, aesthetic appreciation probably goes beyond correctness, that is to say, people do not attend concerts to hear ‘correct’ performances, but to enjoy them. In line with findings about voice attractiveness, one might expect that appreciation of singing would depend heavily on the acoustic properties of voices/singing performances. On the other hand, literature on aesthetic responses to artistic performances (music as well as visual) supports idiosyncrasies on the listener’s side.

Here we aimed to quantify how much the aesthetic appreciation of singing voices depends on objective properties of the sung stimuli and to investigate which acoustic qualities—and their perceptual counterparts—are relevant in this process. To this end, we collected liking ratings (online and lab experiments) and perceptual ratings (lab experiment) of short excerpts of a cappella singing, and we examined, through statistical modeling, the relationship between liking ratings, acoustic properties, and perceptual ratings of these singing performances.

## Results

### Large variability in liking of singing performances

**Figure 1:**
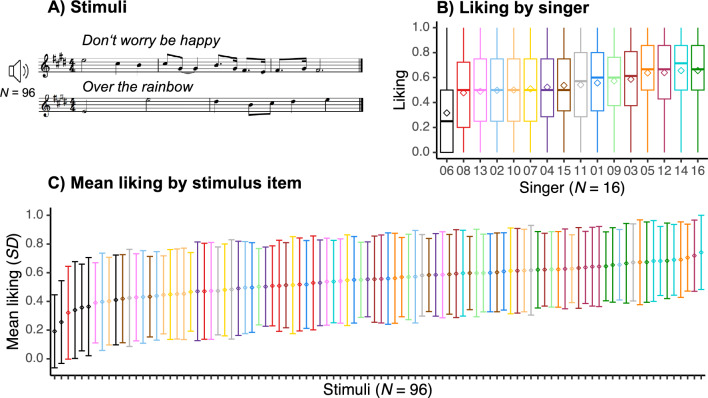
Online experiment. (**A**) Methods: 326 participants rated on a scale of 1 to 9 how much they liked short excerpts of two different melodies, performed at different times and without lyrics, by 16 trained female singers. (**B**) Boxplots of (within-participant normalized) liking ratings by singer. Colors correspond to individual singers. Diamonds depict average liking ratings. Lower and upper hinges correspond to the first and third quartiles, and whiskers extend from the hinge to 1.5 * inter-quartile range (Tukey-style boxplot). (**C**) Mean (within-participant normalized) liking ratings ranked from least to most liked stimuli. Each stimulus was rated by 103, 106 or 117 participants. As in Fig. 1b, colors correspond to individual singers. Diamonds depict average liking ratings per stimulus item, and error bars correspond to one standard deviation above and below the average value.

In a short online experiment (~ 12 min long), 326 participants rated on a 9-point scale how much they liked excerpts of two contrasting a cappella melodies (see Fig. 1A and "Methods" section for details), performed repeatedly by 16 highly trained female singers, for a total of 96 stimuli. Each stimulus was rated by 103, 106 or 117 participants. Despite the large variability in liking ratings, preferences for some stimuli emerged (Fig. 1C), with, e.g., low ratings for the performances of Singer 6 and higher ratings for the performances of Singer 16 (Fig. 1B).

To better understand the large variability of liking ratings observed in Fig. 1, we examined participants’ intra- and inter-rater reliability. The consistency of ratings in a subset of 16 repeated trials revealed a mean test–retest correlation of 0.41 (*SD* = 0.29) and a distribution ranging from − 0.61 to 0.95. We computed Krippendorff’s alpha (α_*K*_) for the (within-participant normalized) liking ratings and found low agreement across participants (α_*K*_ = 0.10, α_*K*_ = 0.07 based on raw ratings). Agreement was higher among 145 “consistent” participants (with *r*_test-retest_ scores higher than 0.5; α_*K*_ = 0.19; α_*K*_ = 0.14 based on raw ratings). Note that this is in line with previous literature reporting variably low agreement between raters in perceptual tasks using music (e.g.:^22,23^ and voice stimuli^24^. Please see Supplementary Figure S1 for the distribution of raw and within-participant normalized liking ratings.

### Very limited role of acoustic features in singing voice preferences

To examine the role of acoustics in singing voice preferences, we constructed linear mixed models that included acoustic features (i.e., acoustic model) as well as features selected from Music Information Retrieval (MIR) and from the Soundgen R package, which was developed to deal with human nonverbal vocalizations^25^. See "Methods" section and Supplementary Information for details about MIR and Soundgen models, Supplementary Table S1 for descriptive statistics of acoustic features by melody and Supplementary Figure S1 for an illustration of the distribution of acoustic features by melody. The acoustic model predicting (within-participant normalized) liking ratings from pitch interval deviation, tempo, energy ratio, harmonics-to-noise ratio, vibrato rate, vibrato extent, jitter, shimmer, and cepstral peak prominence (CPP), and including random intercepts for participants as well as for stimulus items nested in singers, performed better than the null model (*χ2* (9) = 23.597, *p* < 0.01). However, the acoustic model predicted remarkably little variance in liking ratings (Supplementary Table S2), with almost all of the modeled variance captured by the random effects (marginal *R*^2^ = 0.016, conditional *R*^2^ = 0.24). Note that fitting the acoustic model to a subset of 145 “consistent” participants (*r*_test-retest_ scores > 0.5) led to similar results, that is, very low prediction based on fixed effects (marginal *R*^2^ = 0.025, conditional *R*^2^ = 0.3). This suggests that the low prediction was not simply a consequence of participants’ inconsistent rating behavior, which could stem from inattention or lack of engagement to the task—common concerns in any experimental task, but especially so in the case of online experiments^26^. When predicting liking ratings from selected features from MIR and from the Soundgen toolbox, we observed that the best performing MIR model and the Soundgen model also could not account for much of the variance in liking ratings (Supplementary Table S3: marginal *R*^2^ = 0.024, conditional *R*^2^ = 0.227 for the best performing MIR model; marginal *R*^2^ = 0.025, conditional *R*^2^ = 0.223 for the Soundgen model). Here again, fitting the same models on a subset of 145 consistent participants also led to low prediction based on fixed effects (marginal *R*^2^ = 0.047, conditional *R*^2^ = 0.281 for the MIR model and marginal *R*^2^ = 0.052, conditional *R*^2^ = 0.273 for the Soundgen model). In summary, the acoustic features under study have been shown to be directly relevant to listeners when evaluating the technical aspects of singing performances (i.e., correctness) and spoken voice attractiveness. However, the modeling approach revealed their very limited role in predicting variance of singing preferences.

### Perceptual features predict variance in singing preferences

**Figure 2:**
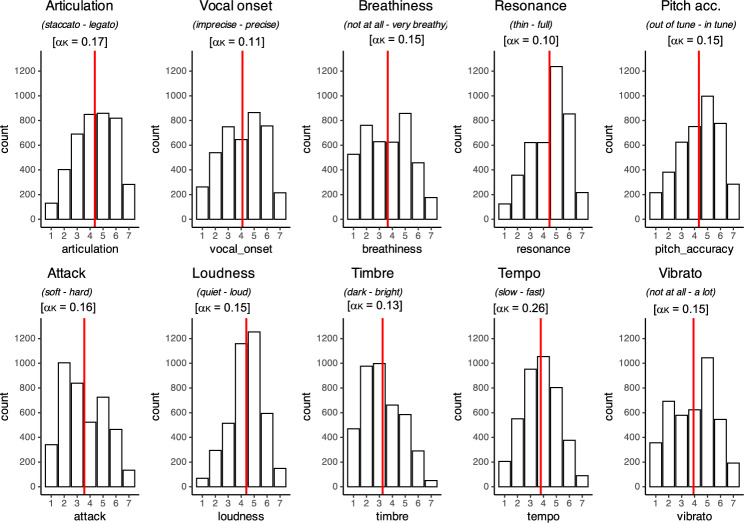
Distribution of perceptual ratings collected in Experiment 2 (*N* = 42 participants), along with anchor words for each bipolar scale. The red line represents the mean rating, and numbers within brackets are Krippendorff’s alpha inter-rater agreement based on within-participant normalized ratings (see Supplementary Table S6 for values of Krippendorff’s alpha based on raw ratings).

To explore how much aesthetic appreciation of singing is grounded in perceptual features of the voices—how listeners perceive acoustic characteristics—we collected ratings of the stimuli on several perceptual features in a lab experiment. Forty-two participants rated the same 96 stimuli on 10 different scales (Fig. 2 and see "Methods" section for details; see Supplementary Figure S3 for the distribution of normalized ratings). They also provided liking ratings, allowing us to replicate the results of the online experiment in a controlled setting. Similar to the online experiment, the distribution of liking ratings was spread and a gradual pattern of average preferences emerged (Supplementary Figure S4b and c). Mean liking ratings per stimulus item correlated highly with the ones from the online experiment (*r*_(94)_ = 0.85, *p* < 0.001; Supplementary Figure S4a). Also in line with the online experiment, fitting the acoustic, MIR and Soundgen models to the data of the lab experiment led to similarly low prediction (Supplementary Tables S4 and S5), that is, in both cases less than 3% of the variance in liking ratings could be explained based on fixed effects, and a larger proportion of variance was captured by random intercepts for participants and stimulus items.

**Figure 3:**
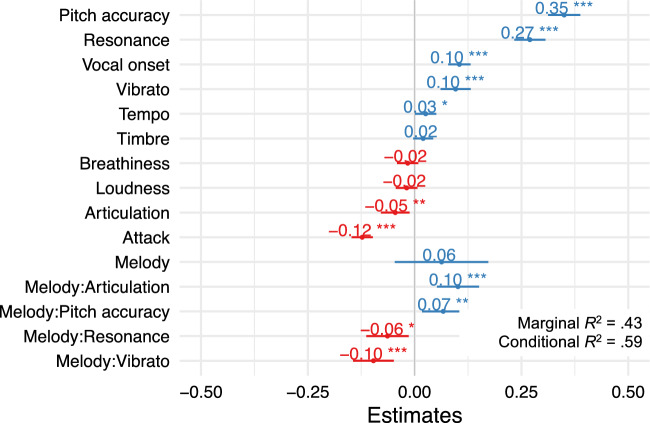
Fixed effects estimates of perceptual model fit on data from Experiment 2 (*N* = 42 participants). All predictors are mean-centered and scaled by one standard deviation. Coefficients and their 95% confidence intervals are plotted. Note the group of reference for melody is *Don’t worry be happy*. ***p < 0.001; **p < 0.01, *p < 0.05.

The perceptual model (Fig. 3 and Supplementary Table S7) accounted for 43.4% of the variance in liking ratings based on fixed effects (marginal *R*^2^ = 0.433, conditional *R*^2^ = 0.588). This model includes as fixed effects the 10 collected perceptual features, melody, interactions between melody and articulation, pitch accuracy, resonance and vibrato; and random intercepts for singer, stimulus items (nested in singer) and participants, as well as random slopes for the effect of melody over participants. The coefficients of the perceptual model show that liking ratings were higher for stimuli perceived as more accurate in terms of pitch, with a full resonance, with some vibrato, with precise and soft vocal onsets and with somewhat fast tempi. There were interactions between melody and some of these features: the effect of pitch accuracy (preference for stimuli perceived as in tune) was more pronounced for the melody *Over the rainbow* (possibly because of the sustained octave jump in the beginning of the melody, making pitch inaccuracies particularly easy to detect). The effect of resonance (preference for “full” resonance) was more pronounced for the melody *Don’t worry be happy*. The effect of vibrato (preference for use of vibrato) was less pronounced and actually not significant for *Over the rainbow*, indicating a preference for use of (some) vibrato only for the *Don’t worry be happy* melody. Regarding the articulation effect (staccato–legato), its interaction with melody indicates preferences in opposite directions depending on the melody: liking ratings were higher as articulation ratings increased (that is, as stimuli were perceived as more legato) for *Over the Rainbow*, and in the opposite direction (that is, as stimuli were perceived as more staccato) for *Don’t worry be happy*. This is an interesting example of participants’ higher-level stylistic conceptions of how each melody should be performed influencing their liking ratings.

### Definition of perceptual features

The discrepancy between the role of perceptual features and the quasi-non-existent role of acoustic features in liking of singing was surprising. To further understand how listeners rate perceptual features, we examined: (1) the inter-rater agreement for each scale; (2) the relationship between the perceptual and acoustic features, independently of the liking ratings; and (3) the role of participants’ characteristics in the use of acoustic and perceptual features.

#### Inter-rater agreement

Inter-rater agreement in participants’ perceptual ratings was low, as shown by Krippendorff’s α (α_K_—see Fig. 2, numbers between brackets, and see Supplementary Table S6 for comparison of α_*K*_ values based on raw and normalized ratings). This is in line with literature reporting variably low agreement in perceptual rating tasks using music stimuli both with lay listeners^22,24^ and experts^23^. See Supplementary Table S6 for intra-class correlation coefficients (ICCs; single random raters, absolute values) as an alternative measure of agreement, to allow for direct comparison with other relevant studies of perceptual ratings of spoken (e.g.,^24^), and singing (e.g.,^23^) voices that also report ICCs. Note that ICC values were similar to α_K_ reported in the main text. We also explored an alternative correlational measure of agreement, “mean-minus-one” (MM1), a leave-one-out type of agreement measure^11^ that measures how much individuals agree with the group (see Supplementary Figure S5). This analysis showed that some participants agree a lot more with the average perceptual evaluation than others.

#### Association between perceptual ratings and acoustic features

Some of the perceptual features are presumably more directly linked to objective properties of the stimuli than others, with timbre (bright–dark) and resonance (thin–full) arguably as the most ‘metaphorical’ judgements participants were asked to make. At the other extreme are pitch accuracy, tempo, and loudness, which are directly related to acoustic measures of pitch interval deviation (relating to *f*_*o*_), speed of performance, and sound intensity, respectively. Indeed, even though inter-rater agreement of perceptual ratings was low, we found correlations between the mean perceptual ratings (averaged within each scale across all participants) and their corresponding acoustic parameters ranging from |.21| to |.68| (Supplementary Table S8). Note that the perceived amount of vibrato correlated with measurements of vibrato extent (that is, measurement of how wide the vibrato was), and not with measurements of vibrato rate (that is, how fast the vibrato was). We also explored individual differences in this association, by computing the same correlations but based on subsets of perceptual ratings by each individual participant (see Supplementary Table S9 for summary statistics of the individual correlation scores, and Supplementary Figure S6). The range of these individual correlations was very wide (e.g., for tempo, they ranged from − 0.20 to 0.63). Importantly, in all cases where we describe an association between mean perceptual ratings and acoustic measurements, there were certain individuals for which correlations with acoustic measurements were higher (or lower) than the overall correlations with mean perceptual ratings. For pitch interval deviation, for instance, the overall correlation with mean perceptual ratings was − 0.20, but for one participant it reached − 0.39. We refer to these individual correlation scores between a participant’s perceptual ratings and the corresponding acoustic measurement as “acoustic sensitivities”, and used these values in exploratory analyses in relation to other participants’ characteristics. Interestingly, we found that participants’ "acoustic sensitivity" correlates with their individual MM1 values in the corresponding scale (i.e., with how much the individual agrees with the group in that particular scale): for loudness: *r*(39) = 0.50; tempo: *r*(39) = 0.56; vibrato: *r*(39) = 0.75; breathiness: *r* = − 0.82 (all ps < 0.001); but for pitch accuracy this did not reach significance: *r*(39) = − 0.18, *p* = . 272). Even though the relationship between acoustic measurements and perception is reportedly not that straightforward (see, for instance,^23,27,28^), it makes sense that participants agreed more with the group perceptual evaluation when their perceptual ratings were more correlated with the corresponding acoustic measurements (or when they were more "correct"). Accordingly, we also found a positive association between participants’ music sophistication, as measured by the Gold-MSI^29^, and participants’ “acoustic sensitivity” for vibrato and breathiness. That is, more musically sophisticated individuals also seem to be more “sensitive” or “objective” when rating the amount of vibrato and the degree of breathiness of singing performances. Considering that lay listeners are sensitive to pitch interval deviation^20^, we expected all participants to rely on pitch interval deviation and thus a limited relationship between pitch accuracy and music sophistication, which appears to be the case, with a correlation not reaching significance—though it is in the expected direction (− 0.19).

#### Individual differences in singing appreciation

**Figure 4:**
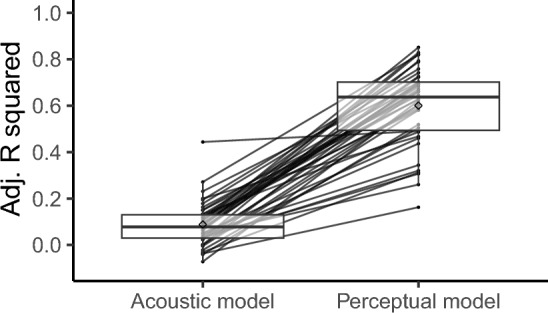
Distribution of adjusted R^2^ values of the acoustic and perceptual models fit individually for each participant of the Experiment 2 (*N* = 42 participants). Lines connect values obtained for each participant; diamonds depict the mean value of each distribution. Lower and upper hinges correspond to the first and third quartiles, and whiskers extend from the hinge to 1.5 * inter-quartile range (Tukey-style boxplot).

To explore individual differences in the role of acoustic and perceptual attributes of the stimuli in singing voice appreciation, we fit individual models for each participant from the lab experiment using multiple linear regression (lm function in R). As before, one model (the acoustic model) includes the acoustic features and one (the perceptual model) includes the perceptual features as predictors. As can be seen in Fig. 4, the resulting adjusted R^2^ values obtained for each participant (referred to here as individual level of prediction) were larger for the perceptual model than the acoustic model (acoustic model: range from − 0.07 to 0.44, *M* = 0.09, *SD* = 0.10; perceptual model: range from 0.16 to 0.85, *M* = 0.6, *SD* = 0.17; paired t-test *t*(41) =  − 19.9, *p* < 0.001).

We also conducted exploratory analyses examining the relationship between the individual level of prediction achieved with the perceptual model (as indicated by the individual adjusted R^2^ values resulting from the previous analysis) and participants’ characteristics. To do so, we fit a multiple regression model predicting the individual level of prediction achieved with the perceptual model—that is, the (Fisher z-transformed) adjusted *R*^2^ values calculated for each participant—from the following participant information, collected via questionnaires: general music sophistication, as measured by the Gold-MSI^29^; the Big-5 personality traits, as measured by the ten-item personality inventory (TIPI;^30^, in a German version^31^); music preferences in terms of the five dimensions of the MUSIC model, as measured by the STOMP-R^32^; participants’ average individual MM1 value (across the 10 perceptual scales); participants’ average “acoustic sensitivity” (across the five selected scales); and participants’ age. The final, reduced model (Supplementary Table S10; see "Methods" section and accompanying .Rmd files for details) indicated that the level of prediction obtained with the perceptual model was higher for more musically sophisticated participants, as well as for older participants; and it was lower for participants with a preference for Mellow music (that is, these participants based their liking ratings less on perceptual attributes of the voices). See Supplementary Table S11 for summary statistics and Supplementary Figure S7 for a correlation matrix of participants’ characteristics.

## Discussion

We investigated singing voice preferences by attempting to separate the relative contribution of objective stimulus features (estimated through acoustic analyses) from (subjective) internal processes on the listener’s side. We asked participants how much they liked singing performances, and modelled liking ratings based on acoustic and perceptual features of the stimuli. We found large individual differences in participants’ preferences, as shown by the highly spread distribution of liking ratings for each stimulus and by the low inter-rater agreement. Nevertheless, even though both experiments differed in terms of setting (online or in the lab) and sampled population (USA-based or Germany-based), we found that mean liking ratings between experiments were highly correlated, suggesting that, at least to some extent, the average preferences that emerged were in fact grounded in attributes of the stimuli.

The modeling analysis showed that the predictors included in the acoustic model, although carefully selected based on previous related research, could not explain the variance in liking ratings. Note that (spoken) voice attractiveness has been strongly linked to acoustic features of the voices—for instance, Babel et al.^16^ could predict 54% of the variance in voice attractiveness ratings based on acoustic parameters (*f*_*o*_, formants, jitter, shitter, CPP, energy and tilt measures) of female voices. In our study, even adding a large number of low-level features from Music Information Retrieval and from the Soundgen package (dedicated to human nonverbal vocalizations) as predictors did not increase the proportion of variance explained. Of course, we did not exhaustively explore all relevant features of the stimuli that might play a role in participants’ aesthetic appreciation, but MIR and Soundgen are assumed to provide a wide range of features, which have been successfully used in different settings (e.g.:^33–39^).

On the other hand, the perceptual model, based on ratings of different perceptual attributes of the voice stimuli, accounted for around 43% of the variance in liking ratings. In other words, the acoustic features themselves did not play a significant role in liking ratings, while the perception that listeners had of acoustic characteristics did. Admittedly, some of the perceptual scales we presented to participants were metaphorical and presumably demanded more top–down processing than others; hence, they could (arguably) be more affected by individual differences in participants’ internal processing (e.g.: expertise) than others. Extreme examples of this would be the scales for resonance (thin–full) and timbre (dark–bright), which demanded a higher level of abstraction than scales directly related to a particular physical dimension, like pitch accuracy (out of tune–in tune), tempo (slow–fast) and loudness (quiet–loud), which relate perceptually to *f*_*o*_ accuracy, speed of performance and sound intensity, respectively.

The associations between acoustic measurements and corresponding average perceptual ratings were between low (r = .− 21 for pitch accuracy) and moderate (r = − 0.61 for breathiness). This is in line with previous research showing variable alignment between acoustic/MIR descriptors and human perceptual ratings^22,23,40,41^. Inspection of individual correlation scores (referred to here as “acoustic sensitivities”) shows a wide range of variation across participants, with some of them showing higher (or lower) individual scores than what is reported for the group as a whole. Exploratory analysis showed that individuals with higher “acoustic sensitivity” also agreed more with the average group evaluation (as measured by individual MM1scores); and that individuals with higher “acoustic sensitivity” for perception of breathiness and vibrato in singing performances scored higher on music sophistication, as measured by the Gold-MSI. These findings highlight the role of individual differences in perception. On the other hand, the limited alignment between perceptual ratings and acoustic estimates may also be related to the limited variance of the acoustic properties themselves in the stimulus set. For instance, the singing performances (by highly trained singers) had remarkably good pitch accuracy: the average pitch interval deviation across all performances was 22 cents (*SD* = 11, min = 5.4, max = 55), which is under the threshold of mistuning perception for a large part of the population^20^. In the case of loudness, to avoid possible confounds of loud performances overly influencing liking of our singing stimuli^42,43^, we loudness normalized all singing performances to the same level of − 14 Loudness Units relative to Full Scale (LUFS). So, similarly as for pitch interval deviation, the narrow range of loudness variation in the stimuli likely limited participants’ performance in detecting these not so drastic differences across stimuli.

We found low inter-rater agreement both in liking ratings of both experiments and in perceptual ratings of Experiment 2. The low inter-rater agreement could, to some extent, be related to the previously mentioned relative homogeneity of the stimulus set: it would be easier for participants to agree in their pitch accuracy ratings if there were big differences in pitch accuracy between performances. The low inter-rater agreement could also potentially be linked to participants’ confusion about what the terms meant, though participants were thoroughly instructed about the definition of each perceptual feature, and studies suggest that untrained listeners are able to evaluate spoken^44^ as well as singing voices^24^ if suitable scales are made available to them. For instance, using the Geneva Voice Perception Scale (GVPS^44^), listeners without any specific training were able to rate (spoken) voices in terms of loudness, pitch, sharpness, intonation, roughness, articulation, among others, though with sizeable differences in reliability estimates across scales (single measure ICCs ranging from 0.31 for roughness to 0.85 for loudness). Merrill^24^ investigated untrained listeners’ abilities to rate singing voices from selected excerpts of popular songs, and reported an average ICC measure of 0.39 for nine relevant features, again with sizeable differences across scales ranging from negative values for pitch (− 0.44) and articulation precision to higher values for noise (0.54) and tension (0.57). Interestingly, the scales introduced by these studies with untrained listeners led to higher inter-rater agreement than what was reported by Lange and Frieler^23^ among experts (sound engineers) rating perceptual features of contrasting music stimuli, with a mean (in the case, also comparable to ICCs) α_K_ of 0.28 for 16 perceptual variables and a mean α_K_ of 0.19 for six emotion-related perceptual variables. Generally, individual differences in the use of (subregions of) scales may also drive some of the lack of consistency across raters (i.e., potentially different profiles of raters, see^22^). We found that normalizing ratings within participant reduced the variance captured by random intercepts in the linear mixed models considerably, though with no impact on the achieved prediction based on fixed effects.

Music preferences are affected by listeners' cultural backgrounds and social influences such as the projection of one's identity^45,46^, and are influenced by age^47^, expertise^48^ and past experience^49^. Further, music preferences depend on and convey personality traits^32,50^, and recent studies have linked psychological traits and preferences for certain low- and high-level musical features^51–53^. In light of this broad literature, we also explored the influence of participants’ characteristics on their use of perceptual features to evaluate singing performances, and found that prediction of liking based on perceptual features was higher among more musically sophisticated and older participants, and lower among participants with a preference for Mellow music (which, according to the MUSIC model^32^, is slow, quiet and not distorted; associated with the music genres soft rock, R&B and adult contemporary; and perceived as romantic, relaxing and not aggressive). One could speculate that the higher prediction of liking based on perceptual features among more musically sophisticated participants might be linked to higher perceptual skills and/or higher reliance on such skills.

One limitation of these brief explorations comes from the relative (musical) homogeneity of our stimulus set, which limits the opportunity for large perceptual differences in the music material (i.e., only two contrasting melodies). Note that this relative homogeneity from a broader musical point of view was a conscious choice on our part, aimed at emphasizing the variability in the voices in the set. We used high quality, enjoyable naturalistic singing performances, with 16 singers providing three takes for each of two contrasting melodies, encouraging participants to focus primarily on perception of the voices themselves rather than the melodies. Additionally, we made the decision not to (attempt to) record amateur or untrained singers due to the challenges associated with satisfactorily performing these melodies a cappella. Including less polished performances could increase the variability in the stimulus set and the generalizability of findings (see^54^ for an example of a large-scale online approach to collect vocal productions cross-culturally and in the general population). In any case, our finding of consistent average preferences for certain singers across two experiments involving participants from two different countries indicates that there was sufficient variability in the stimulus set to elicit considerable preferences. One further limitation of our stimulus set is that both melodies were likely well known to participants. As an example, the interaction between melody and articulation ratings (indicating an overall preference for staccato performances for the melody Don’t worry be happy, and for legato performances for the melody Over the rainbow) suggests that participants had certain expectations on how these melodies should be performed. Familiarity effects^49^ may have influenced participants’ enjoyment of performances in different and unexpected ways. In future studies, it would be desirable to investigate aesthetic preferences with a more varied stimulus set and using novel melodies to control for such familiarity effects, and to increase the generalizability of findings.

While the aesthetic appreciation of singing performances can certainly be approached from the perspective of their musical features, it is also subject to specificities of the voice as a special type of stimulus: beyond supporting speech in interpersonal communication, a person’s voice can also indicate personality traits^55–57^ and emotional states^58^. The conceptual structure of the aesthetic appreciation of voices differs from conceptual representations underlying other domains of aesthetics^59^: the category of beauty is also prevalent, but not among the most frequent adjectives used; and the three dimensions determinant of voice preferences described in that study—pitch (deep/high), loudness (quiet/loud) and vocal quality (soft/rough)—were not found in studies on the conceptual structure of aesthetics of objects, music or visual arts. Considering the wider role of the voice as a sociobiological signal, it would be interesting to further explore the similarities and specificities of the aesthetic appreciation of spoken and singing voice in an integrated way. For instance, Valentova et al.^60^ reported high correlations between attractiveness ratings of matched spoken and sung voices by naïve participants singing excerpts of *Happy birthday* and national anthems, and even argued that singing and speaking may serve as redundant “backup signals”, both cuing body traits and reproductive fitness. Further studies could expand these finding by investigating the aesthetic appeal of a broad range of contrasting but matched vocalizations (i.e., produced by the same voices).

The famous saying “beauty is in the eye of the beholder”—or perhaps “beauty is in the auditory system of the listener”—illustrates a long-standing debate about the relative contribution to aesthetic appraisals of stimulus features versus processing on the listener’s side. Our finding that mean liking ratings correlated highly between participant samples from Germany and the USA hints at cross-cultural average preferences, presumably based (to some extent) on attributes of the stimuli. However, one should keep in mind that both these samples come from Western, educated, industrialized, rich, and democratic (WEIRD^61^) nations. It would be desirable to investigate singing voice preferences in other populations, ideally including non-WEIRD populations (as well as using stimuli material in other styles and/or from multiple cultures to increase the generalizability of findings). So far, our results suggest that, in the case of the singing voice, preferences depend substantially on both attributes of the performances and on listeners’ characteristics, but this relationship is a complex one: liking ratings do not rely on acoustic features of the voices per se but on how the voices are perceived by participants.

## Materials and methods

### Material used in both experiments

#### Recordings of pop singing

##### Singers

Sixteen highly trained female singers whose age ranged from 19 to 56 years old, with an average of 26.18 (*SD* = 9.63), were invited at the Berklee College of Music in Boston, USA. The average length of vocal training among participants was 9.47 years (range: 1 to 29 years, *SD* = 7.12), while the average length of musical instruments training was 11.77 years (range: 0 to 30 years, *SD* = 7.8).

##### Selected songs

Each singer was asked to perform the songs *Don’t Worry Be Happy*, by Bobby McFerrin, and *Over the Rainbow*, by Harold Arlen, without lyrics and in a /u/ sound. Each singer recorded between four and seven takes of each melody. For each melody, we selected three takes from each of the 16 singers for acoustic analysis and subsequent use in the experiment. The stimulus material thus comprised 48 excerpts of each melody, for a total of 96 stimuli, all lasting between 6 and 9 s.

##### Recording procedure

After receiving instructions and signing the consent form, singers were invited to warm up their voices for 3 to 5 min. They were then asked to sing the theme of *Don’t Worry Be Happy* (which is whistled in the original song—Fig. 1A) five times. They were then asked to sing the first phrase of the refrain of *Over the Rainbow* five times (Fig. 1A). The starting pitch height (E5 or E4) was played before each recording. Recordings took place in a sound attenuated booth, using a Neumann (U87) microphone placed at approximately one meter in front of the singer’s mouth; with 16 bits per sample and 48 kHz sampling rate.

##### Audio processing and stimulus selection

For each singer, we selected three takes of each melody for further processing and use in the experiment. Stimuli were cut using Audacity software (version 2.3.3). All stimuli were normalized to the same loudness level of -14LUFS according to the EBU-R128 standards with the software To Audio Converter (Version 1.0.14(902)).

##### Acoustic analyses

The procedures to segment performances into individual notes and calculate vibrato rate, vibrato extent and energy distribution are extensively described in^19,62,63^ and are described succinctly here. Using AudioSculpt 2.9.4v3 software (IRCAM, Paris, France), we first manually placed markers on boundaries of individual notes and used this information to segment each singing performance into individual notes with a sox script. Acoustic analysis was then conducted based on individual notes. Using OpenMusic 6.3 software (IRCAM, Paris, France), we focused on one note per performance (the last note of the *Don’t Worry Be Happy* excerpt, a F3 sharp, and the first note of *Over the Rainbow* excerpt, an E3) to calculate vibrato rate, vibrato extent and energy distribution. Vibrato rate corresponds to the number of quasiperiodic modulations of the *f*_*o*_ per second (in Hz). Vibrato extent corresponds to the amplitude of the *f*_*o*_ variations within the same tone (in cents). The energy distribution measure is the ratio of the energy of the 2.4–5.4 kHz band divided by the energy across the 0–10 kHz band—the rationale behind this computation is that a high score in the energy distribution variable indicates a strong reinforcement of the band containing the singer’s formant. We used Praat^64^ (Version 6.0.46), to extract: mean *f*_*o*_; jitter (local), the perturbation in the *f*_*o*_ from cycle to cycle; and shimmer (local), the perturbation in the amplitude of the *f*_*o*_ from cycle to cycle. Additionally, features relevant to the description of spoken voice were extracted using VoiceSauce^65^, following Babel et al.^16^ and Bruckert et al.^17^: spectral tilt, a measure of voice quality, where higher values of tilt indicate breathier voices and lower values of tilt indicate creakiness^66^. We computed the amplitude of the first harmonic minus the amplitude of the second harmonic (H1–H2), and the longer distance measure of the first harmonic minus the peak amplitude of the first, second, and third formants (H1–A1, H1–A2 and H1–A3, respectively, both corrected and uncorrected, exploratorily). We also extracted the Harmonic-to-Noise ratio (the ratio between periodic and non-periodic components of a speech sound; the higher the HNR, the less hoarse a voice sounds^67^ in the 0–3.5 kHz range (HNR35); and the Cepstral Peak Prominence (CPP), a measure of breathiness and overall dysphonia^68^. To measure how accurate performances were in terms of pitch, based on mean *f*_*o*_ of each individual note, we converted *f*_*o*_ values from Herz to cents (100 cents corresponds to one semitone; the reference lowest note used was 261.626 Hz); and then computed successive pitch interval deviations by subtracting the *f*_*o*_ of adjacent notes and comparing values to “correct” pitch intervals (i.e., according to musical notation). For example, if the interval between two successive notes of a melody should form a major second (which corresponds to an interval of 200 cents), and a singer made a quarter-tone mistake, i.e. if she sang an interval of 150 or 250 cents, we considered that the error was 50 cents in relation to the expected interval. We finally averaged the pitch interval deviation per performance. This procedure has been used and validated in several previous studies^19,21,62,69^. Note that we noticed imprecision in estimates of mean *f*_*o*_ for some very short notes, so we excluded the fourth note of Over the rainbow and the seventh note of Don’t worry be happy from pitch interval deviation computations. Please see Supplementary Figure S8 for a demonstration of this. For jitter and shimmer, we noticed numerous aberrant values for very short notes, indicating measurement imprecision on such short notes, so we trimmed values higher than two standard deviations above the mean value (for each of these two features). This meant excluding 5% of all measurements for shimmer and 2.86% for jitter. We also computed average tempo of each performance as the excerpt’s length divided by the number of beats. A summary of the acoustic properties of the voice stimuli used in the study is presented in Supplementary Table S1 (also see Supplementary Figure S2 for boxplots of these measures). A correlation matrix of acoustic features included in the acoustic model and liking ratings from both experiments is reported in Supplementary Figure S9.

##### Batch extraction of further acoustic/MIR features

To further characterize our stimulus set and based on whole performances, we also analyzed our stimuli using features from Soundgen^25^, an open-source toolbox for voice synthesis, manipulation, and analysis, published as a library (package) for the R programming language; MIRToolbox^70^, a Matlab toolbox for music information retrieval (MIR); and Essentia^71^, an open-source C +  + library for music information retrieval. From Soundgen, we used the analyze function to batch extract several acoustic features. From the output of 156 features (presented as mean, median, and standard deviation summaries per file), we excluded median statistics and voiced_ alternatives because they were highly correlated with their equivalent mean statistics and general correspondent measures, respectively. This led to a core subset of 54 features: amEnvDep_mean, amEnvDep_sd, amEnvFreq_mean, amEnvFreq_sd, amMsFreq_mean, amMsFreq_sd, amMsPurity_mean, amMsPurity_sd, ampl_mean, ampl_sd, CPP_mean, CPP_sd, dom_mean, dom_sd, entropy_mean, entropy_sd, entropySh_mean, entropySh_sd, flux_mean, flux_sd, fmDep_mean, fmDep_sd, fmFreq_mean, fmFreq_sd, harmEnergy_mean, harmEnergy_sd, harmHeight_mean, harmHeight_sd, HNR_mean, HNR_sd, loudness_mean, loudness_sd, novelty_mean, novelty_sd, peakFreq_mean, peakFreq_sd, pitch_mean, pitch_sd, quartile25_mean, quartile25_sd, quartile50_mean, quartile50_sd, quartile75_mean, quartile75_sd, roughness_mean, roughness_sd, specCentroid_mean, specCentroid_sd, specSlope_mean, specSlope_sd, subDep_mean, subDep_sd, subRatio_mean, subRatio_sd. For the two other libraries of MIR features (MIRToolbox and Essentia), we focused on low-level features, since higher-level features would not vary significantly across our two types of short a cappella melody excerpts. From the MIRToolbox, we extracted the following features: low_energy, spec_entropy, brightness, flatness, zerocross, pitch, roughness, mirtempo, rolloff85, rms, rolloff95, spread, skewness, regularity, flux_med, keyclarity, flux, kurtosis, mode, subband (1–10), mfcc (1–10), centroid, pulse_clarity, harmonic_change, fluctuation_max, spectral_novelty. Note that subband and mfcc refer to 10 spectral subbands. For each feature, an average value and standard deviation were extracted, adding up to a total of 80 variables (since for some std variables extraction failed, as well as for mfcc1_mean and mfcc_std). From Essentia, we used the out-of-box executable streaming extractor freesound (https://essentia.upf.edu/freesound_extractor.html) to extract the following low-level features: average_loudness, barkbands_kurtosis, barkbands_skewness, barkbands_spread, dissonance, hfc, pitch, pitch_instantaneous_confidence, pitch_salience, silence_rate_20dB, silence_rate_30dB, silence_rate_60dB, spectral_complexity, spectral_crest, spectral_decrease, spectral_energy, spectral_energyband_high, spectral_energyband_low, spectral_energyband_middle_high, spectral_energyband_middle_low, spectral_entropy, spectral_flatness_db, spectral_flux, spectral_rms, spectral_rolloff, spectral_skewness, spectral_spread, spectral_centroid, spectral_kurtosis, spectral_strongpeak, zerocrossingrate, barkbands (01–27), frequency_bands (01–27), gfcc (01–13), mfcc (01–13), scvalleys (01–06), spectral_contrast (01–06). Note that barkbands and frequency_bands refer to 27 spectral subbands, gfcc and mfcc refer to 13 spectral subbands, and scvalleys and spectral_contrast to six subbands. For each feature, an average value and standard deviation were extracted, and in some cases also the variance of the derivative of a feature (dvar), adding up to a total of 327 features. Note that features were highly correlated with each other: see Supplementary Information (Supplementary Methods: Selecting features from Music Information Retrieval) and accompanying .Rmd files for details and strategies used to subset features and subsequently model liking ratings based on Soundgen and MIR features*.*

### Methods specific to experiment 1

#### Participants

Recruited from Amazon Mechanical Turk (https://www.mturk.com), a total of 330 participants (180 male, 147 female, 3 undisclosed, *M* = 38.3 years old, *SD* = 11.6) completed the whole experiment online. All participants provided informed consent in accordance with the Max Planck Society Ethics Council approved protocol (application 2018–38) and were paid at a US $9/hour rate according to how much of the experiment they completed.

#### Procedure

Experiment 1 was designed and implemented using PsyNet (https://www.psynet.dev), a Python package for performing complex online behavioral experiments at large scale^72^. PsyNet is based on the Dallinger framework (https://dallinger.readthedocs.io) for hosting and deploying experiments. Participants interact with the experiment via a web browser, which communicates with a back-end Python server cluster responsible for organizing the experiment and communicating with participants. The server cluster was provisioned using Heroku.

##### Audio pre-screening task

Before starting the rating task, participants completed an audio pre-screening task^73^ to ensure they were wearing headphones and could perceive subtle sound differences. This three-alternative forced-choice task consisted in identifying the quietest of three tones. These tones are constructed to elicit a phase cancellation effect, such that when played on loudspeakers, the order of quietness changes, causing the participant to respond incorrectly. Participants passed the test only after answering correctly to at least four of the six trials.

##### Liking rating task

Participants were asked to rate how much they liked singing performances. In each trial, a melody excerpt was played, followed by a screen with the question: How much did you like this performance? Participants responded with a mouse on a graphic display of a scale ranging from 1 (not at all) to 9 (a lot). The next trial began immediately after this response. Each participant rated two blocks of performances, one for each melody, in counterbalanced order. Each block was composed of 16 performances of one melody, each performed by a different singer, followed by 8 repetitions, that were used exclusively in a test–retest consistency analysis.

##### Participants’ information

After the two blocks of trials, participants answered 15 questions from a shortened scale of General Music Sophistication of the Goldsmiths Music Sophistication Index, (items: AE_01, AE_02, EM_04, MT_02, MT_03, MT_06, MT_07, PA_04, PA_08, SA_01, SA_02, SA_03, SA_04, SA_05, SA_06)^29^. Finally**,** participants were asked whether they liked the experiment (open field answers). Answers were mostly positive, with 95.8% of the participants answering “Yes”, 0.9% answering “No”, 0.9% answering some type of mixed response one could summarize as “partially”, and 2.4% providing no answer.

### Statistical analyses

All analyses were performed using R Statistical Software (version 4.1.2)^74^ and R Studio (version 2023.06.0 + 421)^75^. Four participants were not included in further analysis (three participants rated all stimuli “1” and one participant rated all stimuli “8” or “9”), leaving a total of 326 participants.

#### Intra-rater agreement

To analyze the consistency of participants’ ratings, we used the 16 stimuli that were presented twice for each participant (8 from each melody), and calculated a Pearson’s correlation score between first and second presentation for each participant.

#### Normalization of ratings

To account for different rating strategies (i.e., different sections of the scale being used by individual participants), we normalized ratings within each participant between 0 and 1. As expected, the original 9-point scale raw ratings and the normalized ratings were highly correlated (*r* = 0.87, *p* < 0.001). Also note that fitting models based on normalized instead of raw ratings only affected the amount of variance explained by random effects (specifically, captured by subjects’ random intercepts), but did not have any meaningful impact on the amount of prediction by the fixed effects: fitting the same acoustic and MIR models to predict raw liking ratings instead of (within-participant) normalized liking ratings produced similar marginal R^2^ values (please see Supplementary Table S12 for this comparison).

#### Inter-rater agreement

In order to examine inter-rater agreement, we used Krippendorff’s alpha (α_*K*_)^76,77^, a generalization of several known reliability indices, where an α_*K*_ = 1 indicates perfect agreement and α_*K*_ = 0 indicates no agreement at all. We used the kripp.alpha function from the irr R package^78^.

#### Predicting liking from acoustic descriptors

Different linear mixed effects analyses were proposed using the lmer function from the lme4 package^79^. For all models reported, residual plots and QQ-curves were visually inspected to make sure there were no deviations from normality or homoscedasticity, and *p*-values were obtained by likelihood ratio tests of the full model with the effect in question against the model without the effect in question. Variance inflation factor (VIF) was under 5 for all predictors kept in the models. Predictors were mean-centered and scaled by one standard deviation before model fitting. Note that the analyses of the online experiment included the 32 trials per subject (16 from each melody) but not the repeated trials, which only served the purpose of consistency analysis. The null model predicted (within-participant normalized) liking ratings and included only random effects (random intercepts for subjects and stimuli items nested in singers). The acoustic model also included as fixed effects predictors shown to be relevant in studies of perception of pitch accuracy^19,21,80^ and voice attractiveness^17^: pitch interval deviation, vibrato extent, vibrato rate, energy ratio, tempo and harmonics-to-noise ratio, jitter, shimmer and the voice quality measure CPP. Note that the tilt measures H1–H2, H1–A1, H1–A2 and H1–A3 were not included in the model due to multicollinearity issues. A second set of linear mixed models, referred to as MIR models, was fit to predict (within-participant normalized) liking ratings from subsets of low-level MIR features, with subjects and stimuli items (nested in singers) as random effects. Since MIR features were highly correlated with each other, we first explored three different approaches to subset them and then fit three linear mixed models based on the resulting subsets of features. We also fit one separate model based on features from the Soundgen toolkit. Please see the Supplementary Information (Supplementary Methods: Selecting features from Music Information Retrieval/ Selecting features from the Soundgen package) and accompanying .Rmd files for details about feature reduction and model selection.

### Method specific to experiment 2

#### Participants

Forty-two participants (26 female, 16 male), aged from 22 to 75 years old (*M* = 36.8, *SD* = 16.1), completed the whole experiment. The experimental procedure was ethically approved by the Ethics Council of the Max Planck Society, and was undertaken with written informed consent of each participant.

#### Procedure

The experiment was implemented in Labvanced^81^. Participants were tested on a computer located in the laboratories of the Max Planck Institute for Empirical Aesthetics in Frankfurt, Germany. The liking rating scale and the perceptual evaluation scales were presented on the same screen, in German, together with the experimental stimuli to rate. As in the online experiment, participants rated how much they liked each singing performance on a scale from 1 (not at all) to 9 (a lot).

##### Perceptual ratings

Particular to Experiment 2 were perceptual ratings: we developed a series of scales relative to perceptual attributes shown to be relevant in the appreciation of singing. Perceptual attributes were rated on bipolar scales ranging from 1 to 7 and displaying contrasting anchor words on each pole. The scales, their bipolar anchors and the definition presented to participants were the following (translated to English here): Pitch accuracy (in tune–out of tune): how precise is each note along the melody—is the performance in-tune or out-of-tune? Loudness (soft–loud): the magnitude of the auditory sensation: is the voice quiet or loud? Tempo (slow–fast): the speed or pace of the performance—is the performance slow or fast? Articulation (staccato–legato): how notes are connected to each other—are notes detached (staccato) or connected (legato)? Breathiness (not at all–a lot): the amount of air flow in the voice—how breathy does the voice sound? Resonance (thin–full): the fullness or reverberation of a voice: how full is the voice? Timbre (dark–bright): the perceived sound quality of the voice: does the voice sound dark or bright? Attack/Voice onset 1 (soft–hard): the way in which a note begins—is the start of notes soft or hard? Voice onset 2 (precise–imprecise): the way in which a note begins—is the start of notes precise? Vibrato (not at all–a lot): A slight and periodic oscillation of the pitch of a sustained note—how much vibrato does the performer use? Please see Supplementary Table S13 for the German version of these scales and instructions used in the experiment. There was also one question about the vocalic sound produced by the singer, because even though they were asked to sing in an /u/ sound, in many cases the produced vowel wasn’t that clear or constant, and we hypothesized this could influence participants’ liking. Therefore, participants also had to indicate what was the dominant vowel sound perceived in each stimulus in a forced choice response. Participants indicated perceiving a /u/ sound in 53.4% of trials; a /a/ sound in 16.2% of trials; a /o/ sound in 10.8% of trials; a /i/ sound in 1.54% of trials; and an unclear vowel sound in 18% of trials. Note that this predictor was later found insignificant during the modeling analysis and dropped from the perceptual model.

The experiment was divided into six blocks (three per melody). Half of the participants started with the three blocks for *Over the Rainbow*, half with the opposite order. Each block comprised 16 trials, corresponding to one take by each of the 16 singers, presented in a randomized order. The order of these three blocks within each melody was counterbalanced across participants. Following the general instructions, we presented the definitions of the scales and five examples they could listen to. For each experimental stimulus, participants could click on the “play” button as many times as they wanted to and listen to the stimulus again as they rated 10 bipolar scales, the scale for liking and the forced-choice question for dominant vowel. The next page was proposed and viable when all scales and the vowel question were completed and participants pressed the “next” button. Half-way through the experiment (i.e., between the 3 blocks of the first and the second melody), participants completed three questionnaires (see below). Participants completed the experiment at their own pace and took between 1.5 and three hours to complete the experiment. They could take a break at any desired moment, and additional breaks were included between blocks of trials.

##### Participants’ information

In addition to providing biographical data, participants completed three questionnaires:

1. The 18-items subscale of Music Sophistication from the Goldsmiths Music Sophistication Index^29^ in German, as computed in the Gold-MSI configurator (https://shiny.gold-msi.org/gmsiconfigurator; items included: AE_01, AE_02, AE_05, AE_07, EM_04, MT_01, MT_02, MT_03, MT_06, MT_07, PA_04, PA_08, SA_01, SA_02, SA_03, SA_04, SA_05, SA_06)
2. The Ten-Item Personality Inventory (TIPI), a short self-report measure of the Big-Five personality domains^30^, in the German version^31^. Each of the five personality dimensions—Extraversion, Agreeableness, Conscientiousness, Emotional stability (or Neuroticism) and Openness to new experiences—was measured by two items, selected from the high and low poles of each domain. Each question presented two central descriptors, and participants had to rate on a scale from 1 (disagree strongly) to 7 (agree strongly) how much those two traits applied to them.
3. The Reviewed Short Test of Music Preference (STOMP-R), a short self-report inventory for musical preferences^32^; see^82^ for a German validation). Rentfrow et al.^32^ suggested a latent 5-factor structure underlying music preferences, which they named the MUSIC model (Mellow, Unpretentious, Sophisticated, Intense and Contemporary). Participants were asked to indicate their preferences for 23 different music genres on a 7 point scale (dislike strongly, dislike moderately, dislike a little, neither like nor dislike, like a little, like moderately, like strongly). To compute each participant’s preference for each dimension (see instructions retrieved from the author’s website: https://www.psd.psychol.cam.ac.uk/projects-measures), we averaged scores within the following genres: Dance/Electronica, New Age, World (Mellow); Pop, Country, Religious (Unpretentious). Blues, Jazz, Bluegrass, Folk, Classical, Gospel, Opera (Sophisticated); Rock, Punk, Alternative, Heavy Metal (Intense); Funk, Rap/hip-hop, Reggae, Soul/R&B (Contemporary). As in^47^, we excluded the genres Soundtrack and Oldies from analysis because they load on different dimensions. We instructed participants to leave blank the genres they did not know.

#### Statistical analyses

##### Normalization of ratings

As in Experiment 1, we normalized all collected ratings within-participant (between 0 and 1) to account for individual differences in the use of subregions of the rating scales. Raw and normalized ratings were highly correlated (r = 0.95 for liking; r > 0.92 for all perceptual scales; please see Supplementary Figure S3 for the distribution of normalized perceptual ratings). Note that also for data from Experiment 2, fitting our models based on raw versus normalized ratings leads to similar prediction based on fixed effects, and only meaningfully impacts the variance captured by random effects (please see Supplementary Table S12 for this comparison).

##### Inter-rater agreement

Once again, we examined inter-rater agreement with α_K_, but we also computed intraclass correlation scores (ICC2 or single random raters, absolute values), using the ICC function in the psych R package^83^, to facilitate comparison with other studies reporting this agreement measure. We also computed “mean-minus-one” agreement. To compute MM1, a Pearson correlation is computed between a given participant’s set of ratings and the average ratings of all other participants. This is done for all participants in the sample. The resulting individual correlations are first converted to z scores (to reduce bias in estimates^84^), averaged and converted back into an r score for easier interpretation of the final MM1 measure. We calculated MM1 agreement for liking and for each of the perceptual scales separately (Supplementary Figure S5), and were not primarily interested in the overall MM1 index per perceptual scale per se, but on the distribution of individual MM1 values (i.e., for each participant): both the individual MM1 scores in each scale and one average value per participant (computed across all 10 perceptual scales) were used in exploratory analyses, in relation to other participant-related data.

##### Relationship between acoustic measurements and perceptual ratings

We computed Pearson correlations between our acoustic measurements (in some cases taken from the toolboxes MIRToolbox, Essentia and/or Soundgen) and the corresponding average perceptual rating (see Supplementary Table S8). Note that for some of the perceptual scales, there is not one clear acoustic correlate. We computed the correlation between our estimates of pitch interval deviation and participants’ ratings of perceived pitch accuracy; estimates of tempo (as measured by beats per minute) and as perceived by participants; estimates of vibrato extent and perceived amount of vibrato; estimates of vibrato rate and perceived amount of vibrato; estimates of loudness_mean (from the Soundgen package) and perceived loudness (note this measure seems more appropriate than Soundgens’ ampl_mean or MIRToolbox’s rms_mean, since it takes into account the sensitivity of human ears to different frequencies); estimates of cepstral peak prominence (CPP; we compared the estimates from VoiceSauce, which were included in our acoustic model, and from Soudgen) and perceived breathiness; and the measure flux_mean (we compared measures from the MIRToolbox, Soundgen and Essentia) and perceived articulation.

##### Individual differences in the relationship between acoustics and perception

We also explored individual differences in the relationships between acoustic measurements and individual participants’ perceptual ratings. To do so, we computed the correlations mentioned above, but based on subsets of perceptual ratings by each individual participant—we refer to these as “acoustic sensitivity”. This is illustrated in Supplementary Figure S6 (and see summary statistics of the individual correlation scores in Supplementary Table S9). Further, we conducted exploratory analyses based on participants’ “acoustic sensitivity”. For this, we focused on the most straightforward correlations from Supplementary Table S8: we selected the correlations between measurements of pitch interval deviation and perceived pitch accuracy; tempo in beats per minute and as perceived by participants; loudness_mean (from Soundgen) and perceived loudness; Vibrato extent and perceived vibrato; and CPP_mean (from VoiceSauce, which is a predictor in our acoustic model) and perceived breathiness. We first performed a Fisher z-transformation on the individual r-scores (to reduce bias in estimates^84^). We then used these values in two exploratory analyses: a) we calculated the correlation between these values (separately for each scale) and participants’ individual MM1 values (also as z-scores), in an effort to understand if individuals with higher “acoustic sensitivity” tend to agree more with the average group evaluation; b) we calculated an average “acoustic sensitivity” value per participant (by averaging values across the five selected scales) and included it as a predictor in a model investigating the level of prediction achieved with the perceptual model (see Role of participants’ characteristics).

##### Predicting liking from acoustic and perceptual features

We performed the same linear mixed effects analyses reported for Experiment 1, that is, we fit the same acoustic and MIR/Soundgen models. In addition, we built a perceptual model predicting liking ratings from the collected perceptual ratings. The final perceptual model includes as fixed effects the 10 collected perceptual features and melody; interactions between melody and articulation, pitch accuracy, resonance and vibrato; and random intercepts for singer, stimuli items (nested in singer) and participants, as well as random slopes for the effect of melody on participants.

##### Role of participants’ characteristics

To investigate individual differences in how much participants based their liking ratings on acoustic and perceptual features of the stimuli, we proposed two (individual) multiple regression models (with the lm function in R) to each participant, using either the predictors from the acoustic or the perceptual model. We refer to the resulting adjusted R^2^ values of these individual multiple regression models as “individual level of prediction” (for each participant). We then used the individual level of prediction based on perceptual features to investigate the role of participants’ characteristics on how much they based their liking ratings on perceptual features of the stimuli. To do so, we entered the individual level of prediction for the perceptual model (that is, the adjusted R^2^ values resulting from individual fitting of perceptual models) as the dependent variable in a multiple regression model where the predictors were participant-related data collected in the questionnaires of general music sophistication, musical preferences, and psychological traits, as well as participants’ age, participants’ average individual MM1 value (across the 10 perceptual scales) and participants’ average “acoustic sensitivity” (across five selected scales—see Individual differences in the relationship between acoustics and perception). To improve model fit, we performed a Fisher z-transformation of the individual adjusted R^2^ values prior to fitting the model. We used stepwise selection to reduce the model (with the step function in R).

### Supplementary Information

Supplementary Information.

## Data Availability

Supplementary information, the raw data from both experiments and analyses code (as .Rmd files), as well as examples of the singing performances, can be found at https://osf.io/ts24m/. The whole set of singing performances can be made available to interested researchers upon request.
