# Supplementary material for: Perceptual (but not acoustic) features predict singing voice preferences
Source: Sci Rep. 2024 Apr 18;14:8977. doi: 10.1038/s41598-024-58924-9 (PMC11026466; doi:10.1038/s41598-024-58924-9)
Supplement: Supplementary file 1 — Supplementary Information. [file 41598_2024_58924_MOESM1_ESM.pdf]

Supplementary Information for
Perceptual (but not acoustic) features predict singing voice preferences.

Camila Bruder, David Poeppel, & Pauline Larrouy-Maestri

*camila.bruder@ae.mpg.de

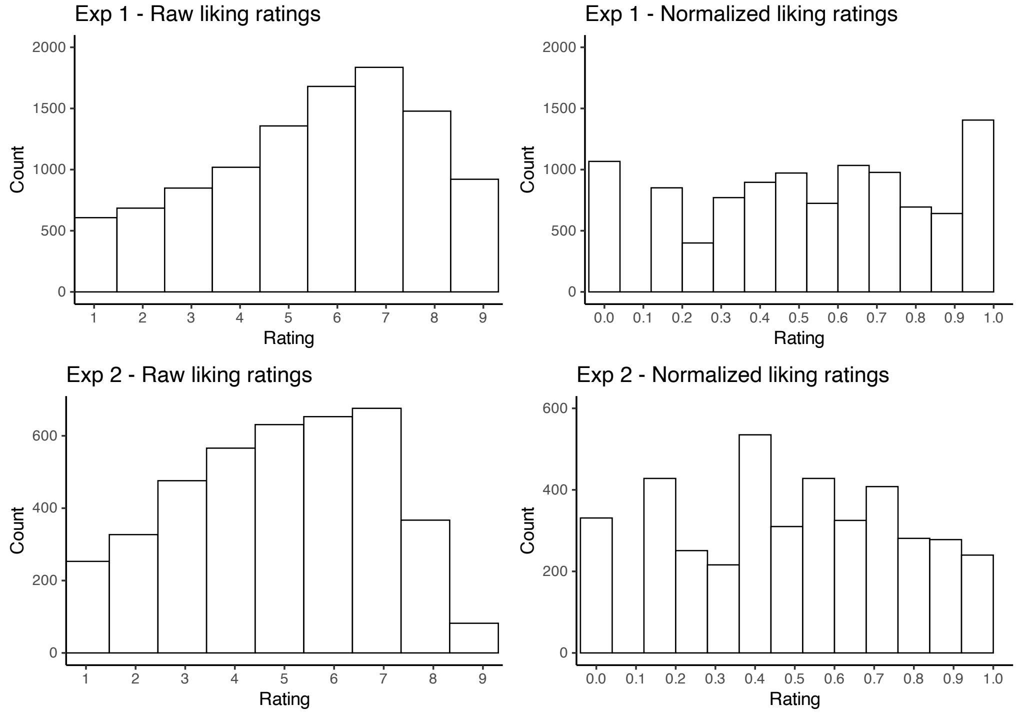

Supplementary Figure S1 : Distribution of raw liking ratings (left) and within-participant normalized liking ratings (right) in Experiments 1 (top) and 2 (bottom).

Supplementary Table S1: Mean, standard deviation, minimum and maximum of the acoustic parameters included in the acoustic model for the 96 singing performances of the two melodies used as stimuli in both experiments. CPP: cepstral peak prominence.

	Dont worry be happy				Over the rainbow			
	**Mean**	**SD**	**Min**	**Max**	**Mean**	**SD**	**Min**	**Max**
**Vibrato extent**	127.67	60.74	16	250	84.08	61.78	4	378
**Vibrato rate**	5.88	0.71	4.25	8.26	6.02	1.4	3.23	10.26
**Pitch interval deviation**	27.74	9.61	10.4	54.8	16.5	8.78	5.4	41
**Tempo**	121.92	10.91	96.4	143.43	72.8	10.19	50.76	94.84
**Energy ratio**	1.8	0.15	1.48	2.1	2.2	0.4	1.59	2.96
**CPP**	20.31	2.29	15.27	24.24	19.82	2.1	16.05	24.64
**Harmonics-to-noise ratio**	61.64	3.52	53.44	68.87	46.18	4.75	31.45	53.42
**Jitter local**	0.0041	0.0011	0.0023	0.0071	0.003	0.0017	0.0013	0.0094
**Shimmer local**	0.036	0.0079	0.022	0.056	0.024	0.0068	0.014	0.04

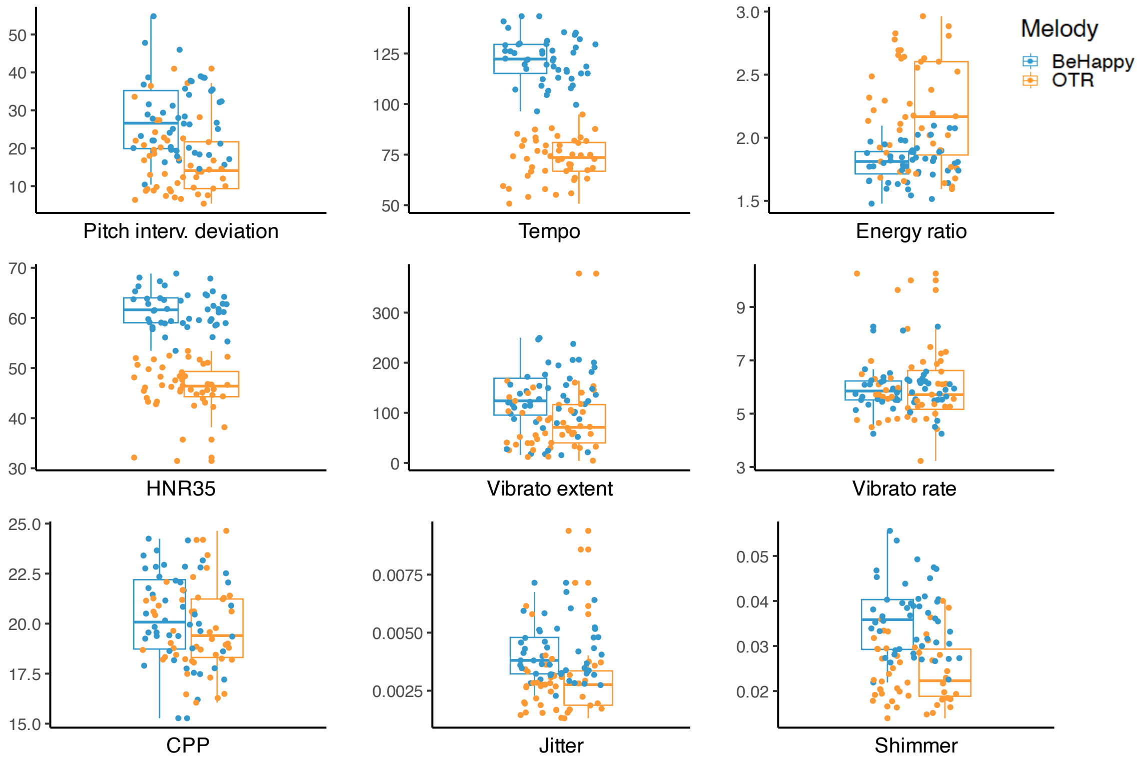

Supplementary Figure S2: Distribution of acoustic features included in the acoustic model. Each dot represents one performance (N = 96), and colors represent the two contrasting melodies, *Don't worry be happy* and *Over the rainbow*. Lower and upper hinges correspond to the first and third quartiles, and whiskers extend from the hinge to 1.5 * inter-quartile range (Tukey-style boxplot). CPP: cepstral peak prominence.

Supplementary Table S2: Fixed effects estimates and model evaluation metrics for the acoustic model fit on data from **Experiment 1** (*N* = 326). 95% confidence intervals are between brackets. All predictors are mean-centered and scaled by one standard deviation. BIC: Bayesian Information Criterion; AIC: Akaike Information Criterion; ICC: Intraclass Correlation Coefficient (indicating proportion of variance captured by random intercepts).

Model syntax: lmer(liking ~ Pitch_interv_deviation + Tempo + Energy + HNR35+ V_extent + V_rate + CPP + Jitter + Shimmer + (1|participant) + (1|singer/item), df1)

	**Acoustic model**
(Intercept)	0.544 ***
	[0.502, 0.585]
Pitch interval deviation	-0.027 **
	[-0.046, -0.009]
Tempo	-0,029
	[-0.059, 0.001]
Energy ratio	0,022
	[-0.003, 0.046]
Harmonics-to-Noise Ratio	0.041 **
	[0.012, 0.070]
Vibrato extent	0,003
	[-0.017, 0.023]
Vibrato rate	0,005
	[-0.011, 0.021]
Cepstral Peak Prominence	-0,011
	[-0.032, 0.011]
Jitter	-0,005
	[-0.026, 0.015]
Shimmer	0,010
	[-0.026, 0.046]
N (trials)	10432
N (participant)	326
N (item:Singer)	96
N (Singer)	16
AIC	3266,799
BIC	3368,335
ICC (participants)	0.127
ICC (item:singer)	0.036
ICC (singer)	0.063
**Marginal R^2**	**0.016**
**Conditional R^2**	**0.238**

*** $p < 0.001$; ** $p < 0.01$; * $p < 0.05$.

Supplementary Table S3: Fixed effects estimates and model evaluation metrics of the proposed MIR and Soundgen models fit on data from Experiment 1 (*N* = 326). All predictors are mean-centered and scaled by one standard deviation. 95% confidence intervals are between brackets. dvar: variance of the derivative; std: standard deviation; dmean: mean of derivative; gfcc: gammatone feature cepstrum coefficients; hfc: high frequency content of a spectrum; amEnvFreq_mean: frequency of amplitude modulation; amEnvDep: depth of amplitude modulation; specCentroid: spectral centroid; entropySh: Shannon entropy; quartile25: 25th. quantiles of the spectrum below the specified cutoff frequency for voiced frames. BIC: Bayesian Information Criterion; AIC: Akaike Information Criterion; ICC: Intraclass Correlation Coefficient (indicating proportion of variance captured by random intercepts). Please see additional information on MIR/Soundgen features and model selection at the Annex in the end of this document.

Model syntax: lmer(liking ~ predictor 1 + predictor 2 + … + (1|participant) + (1|singer/item), df1)

	CorrSelect	Corr_Reduction	Hclust	Soundgen
(Intercept)	0.544 ***	0.544 ***	0.544 ***	0.544 ***
	[0.507, 0.581]	[0.504, 0.583]	[0.506, 0.581]	[0.509, 0.579]
frequency_bands.dvar_13	-0.015 *			
	[-0.029, -0.001]			
brightness_mean	-0.038 ***			
	[-0.054, -0.022]			
regularity_mean	-0.015 *			
	[-0.029, -0.001]			
mode_mean	-0.023 **	-0.024 **		
	[-0.037, -0.010]	[-0.038, -0.009]		
frequency_bands.dmean_15		-0.017 *		
		[-0.030, -0.003]		
hfc.dmean		-0.021 **		
		[-0.034, -0.008]		
spectral_strongpeak.dmean			-0.027 **	
			[-0.044, -0.011]	
dissonance.dmean			0.028 **	
			[0.011, 0.045]	
gfcc.dmean_02			-0.023 *	
			[-0.040, -0.005]	
amEnvFreq_mean				0.036 ***
				[0.019, 0.052]
amEnvDep_mean				0.021 *
				[0.002, 0.041]
specCentroid_mean				0.033 *
				[0.002, 0.064]
entropySh_mean				-0.042 **
				[-0.070, -0.015]
quartile25_sd				-0.025 **
				[-0.040, -0.010]
N (trials)	10432	10432	10432	10432
N (participant)	326	326	326	326

N (Singer)	16	16	16	16
AIC	3208.477	3208.279	3207.814	3215.974
BIC	3273.750	3266.300	3265.835	3288.501
ICC (participant)	0.13	0.129	0.129	0.130
ICC (item:singer)	0.029	0.031	0.033	0.031
ICC (singer)	0.049	0.057	0.049	0.042
**Marginal R^2**	**0.024**	**0.015**	**0.018**	**0.025**
**Conditional R^2**	**0.227**	**0.229**	**0.225**	**0.223**

*** $p < 0.001$; ** $p < 0.01$; * $p < 0.05$.

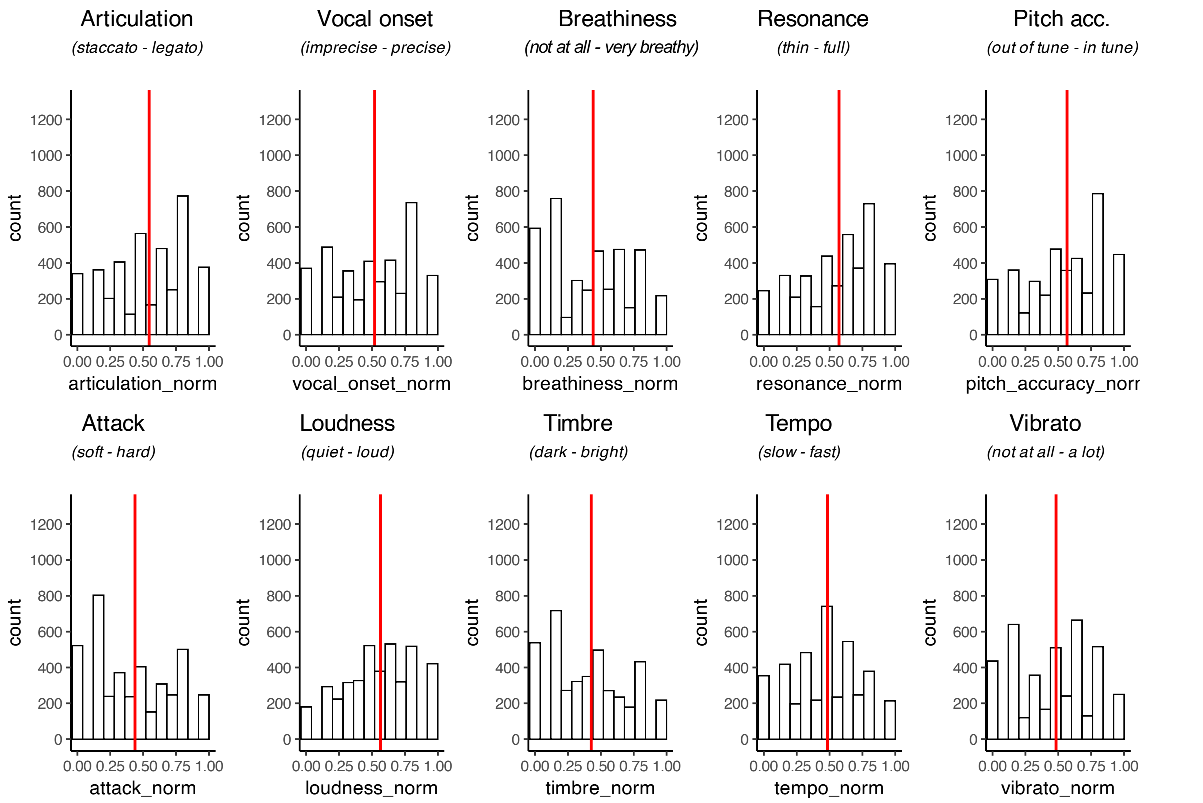

Supplementary Figure S3: Distribution of within-participant normalized perceptual ratings in Experiment 2. The red line represents the mean rating.

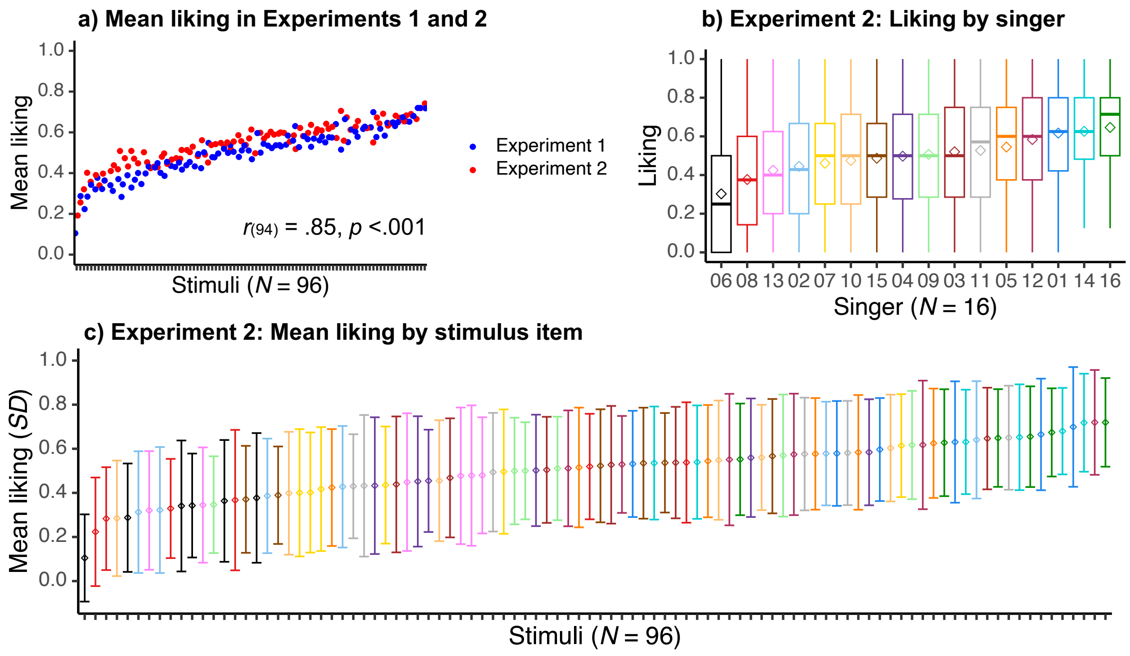

Supplementary Figure S4: Liking ratings from Experiment 2 (*N* = 42) participants replicate the pattern found in Experiment 1. a) Relationship between (within-participant normalized) mean liking ratings from Experiments 1 and 2. b) Boxplots of (within-participant normalized) liking ratings by singer. Colors correspond to individual singers. Diamonds depict average liking ratings. Lower and upper hinges correspond to the first and third quartiles, and whiskers extend from the hinge to 1.5 * inter-quartile range (Tukey-style boxplot). c) Mean (within-participant normalized) liking ratings ranked from least to most liked stimuli. Each stimulus was rated by 42 participants. As in Fig. 1B, colors correspond to individual singers. Diamonds depict average liking ratings per stimulus item, and error bars correspond to one standard deviation above and below the average value.

Supplementary Table S4: Fixed effects estimates and model evaluation metrics for the acoustic model fit on data from Experiment 2 (*N* = 42 participants). 95% confidence intervals are between brackets. All predictors are mean-centered and scaled by one standard deviation. BIC: Bayesian Information Criterion; AIC: Akaike Information Criterion; ICC: Intraclass Correlation Coefficient (indicating proportion of variance captured by random intercepts).

Model syntax: lmer(liking ~ Pitch_interv_deviation + Tempo + Energy + HNR35+ V_extent + V_rate + CPP + Jitter + Shimmer + (1|participant) + (1|singer/item), df2)

	**Acoustic model**
(Intercept)	0.503 ***
	[0.450, 0.555]
Pitch interval deviation	-0.029 *
	[-0.053, -0.005]
Tempo	-0.032
	[-0.071, 0.006]
Energy ratio	0.027
	[-0.005, 0.058]
Harmonics-to-Noise Ratio	0.053 **
	[0.016, 0.090]
Vibrato extent	0.004
	[-0.021, 0.030]
Vibrato rate	-0.002
	[-0.023, 0.019]
Cepstral Peak Prominence	-0.035 *
	[-0.062, -0.008]
Jitter	-0.011
	[-0.037, 0.016]
Shimmer	0.027
	[-0.018, 0.073]
N (trials)	4031
N (item:singer)	96
N (participant)	42
N (singer)	16
AIC	244.927
BIC	333.152
ICC (participants)	0.129
ICC (item:singer)	0.072
ICC (singer)	0.081
**Marginal R^2**	**0.032**
**Conditional R^2**	**0.304**

*** p < 0.001; ** p < 0.01; * p < 0.05.

Supplementary Table S5: Fixed effects estimates and model evaluation metrics of the proposed MIR models fit on data from Experiment 2 (*N* = 42) participants. All predictors are mean-centered and scaled by one standard deviation. 95% confidence intervals are between brackets. dvar: variance of the derivative; std: standard deviation; dmean: mean of derivative; gfcc: gammatone feature cepstrum coefficients; hfc: high frequency content of a spectrum; amEnvFreq_mean: frequency of amplitude modulation; amEnvDep: depth of amplitude modulation; specCentroid: spectral centroid; entropySh: Shannon entropy; quartile25: 25th. quantiles of the spectrum below the specified cutoff frequency for voiced frames. BIC: Bayesian Information Criterion; AIC: Akaike Information Criterion; ICC: Intraclass Correlation Coefficient (indicating proportion of variance captured by random intercepts).

Model syntax: lmer(liking ~ predictor 1 + predictor 2 + … + (1 | participant) + (1 | singer/item), df2)

	CorrSelect	Corr_Reduction	Hclust	Soundgen
(Intercept)	0.503 ***	0.503 ***	0.503 ***	0.503 ***
	[0.453, 0.553]	[0.453, 0.553]	[0.450, 0.555]	[0.456, 0.550]
frequency_bands.dvar_13	-0.008			
	[-0.027, 0.011]			
brightness_mean	-0.032 **			
	[-0.054, -0.010]			
regularity_mean	-0.027 **			
	[-0.046, -0.007]			
mode_mean	-0.022 *		-0.021 *	
	[-0.041, -0.003]		[-0.041, -0.001]	
spectral_strongpeak.dmean		-0.024 *		
		[-0.047, -0.001]		
dissonance.dmean		0.037 **		
		[0.013, 0.060]		
gfcc.dmean_02		-0.015		
		[-0.038, 0.009]		
frequency_bands.dmean_15			-0.022 *	
			[-0.041, -0.003]	
hfc.dmean			-0.007	
			[-0.026, 0.011]	
amEnvFreq_mean				0.028 *
				[0.005, 0.051]
amEnvDep_mean				0.040 **
				[0.014, 0.067]
specCentroid_mean				0.031
				[-0.010, 0.071]
entropySh_mean				-0.044 *
				[-0.081, -0.007]
quartile25_sd				-0.022 *
				[-0.044, -0.001]
N (trials)	4031	4031	4031	4031
N (participant)	42	42	42	42

N (item:singer)	16	16	16	16
AIC	203.388	199.441	201.846	210.846
BIC	260.104	249.855	252.260	273.863
ICC (participant)	0.13	0.128	0.13	0.077
ICC (item:singer)	0.071	0.074	0.076	0.132
ICC (singer)	0.071	0.081	0.069	0.055
**Marginal R^2**	**0.028**	**0.019**	**0.014**	**0.030**
**Conditional R^2**	**0.292**	**0.289**	**0.293**	**0.286**

*** $p < 0.001$; ** $p < 0.01$; * $p < 0.05$.

Supplementary Table S6: Krippendorff's α (α_K) and Intra-class correlations (ICCs; single random raters, absolute values) of ratings collected in Experiment 2 (*N* = 42), computed based on raw or (within-participant normalized) ratings.

	**α_K based on**		**ICC based on**	
**Feature**	**Raw rating**	**Norm rating**	**Raw rating**	**Norm rating**
Articulation	0.18	0.17	0.20	0.18
Attack	0.16	0.16	0.17	0.17
Loudness	0.14	0.15	0.15	0.16
Breathiness	0.14	0.15	0.15	0.16
Pitch accuracy	0.14	0.15	0.16	0.17
Resonance	0.09	0.10	0.10	0.12
Tempo	0.25	0.26	0.27	0.27
Timbre	0.12	0.13	0.13	0.13
Vibrato	0.13	0.15	0.14	0.15
Vocal onset	0.10	0.11	0.11	0.12
**Average**	**0.13**	**0.13**	**0.14**	**0.14**
Liking	0.13	0.16	0.14	0.17

Supplementary Table S7: Fixed effects estimates and model evaluation metrics of the perceptual model fit on data from Experiment 2 (*N* = 42) participants. Note coefficients presented in this table are redundant with the model plot presented in Fig. 3 and are included here to allow for easier comparison with other modeling results. ICC: Intraclass Correlation Coefficient (indicating proportion of variance captured by random intercepts - but note random slopes were specified for the effect of melody over participants).

Model syntax: lmer(liking ~ pitch_accuracy + resonance + vocal_onset + vibrato +tempo + timbre + breathiness + loudness + articulation + attack +melody + melody:articulation + melody:pitch_accuracy + melody:resonance + melody:vibrato + (1 + melody | participant) + (1 | Singer/name), df2)

	**Perceptual model**
(Intercept)	0.116 ***
Pitch accuracy	0.343 ***
Resonance	0.274 ***
Vocal onset	0.100 ***
Vibrato	0.093 ***
Tempo	0.027 *
Timbre	0.020
Breathiness	-0.015
Loudness	-0.020
Articulation	-0.044 **
Attack	-0.115 ***
Melody (ref. Over the Rainbow)	0.010
Articulation:Melody	0.098 ***
Pitch accuracy:Melody	0.065 **
Resonance:Melody	-0.065 *
Vibrato:Melody	-0.094 ***
N (trials)	4031
N (item:singer)	96
N (participant)	42
N (singer)	16
AIC	-2018.457
BIC	-1879.818
ICC (participant)	0.257
ICC (item:singer)	0.025
ICC (singer)	0.015
**Marginal R^2**	**0.433**
**Conditional R^2**	**0.587**

*** p < 0.001; ** p < 0.01; * p < 0.05.

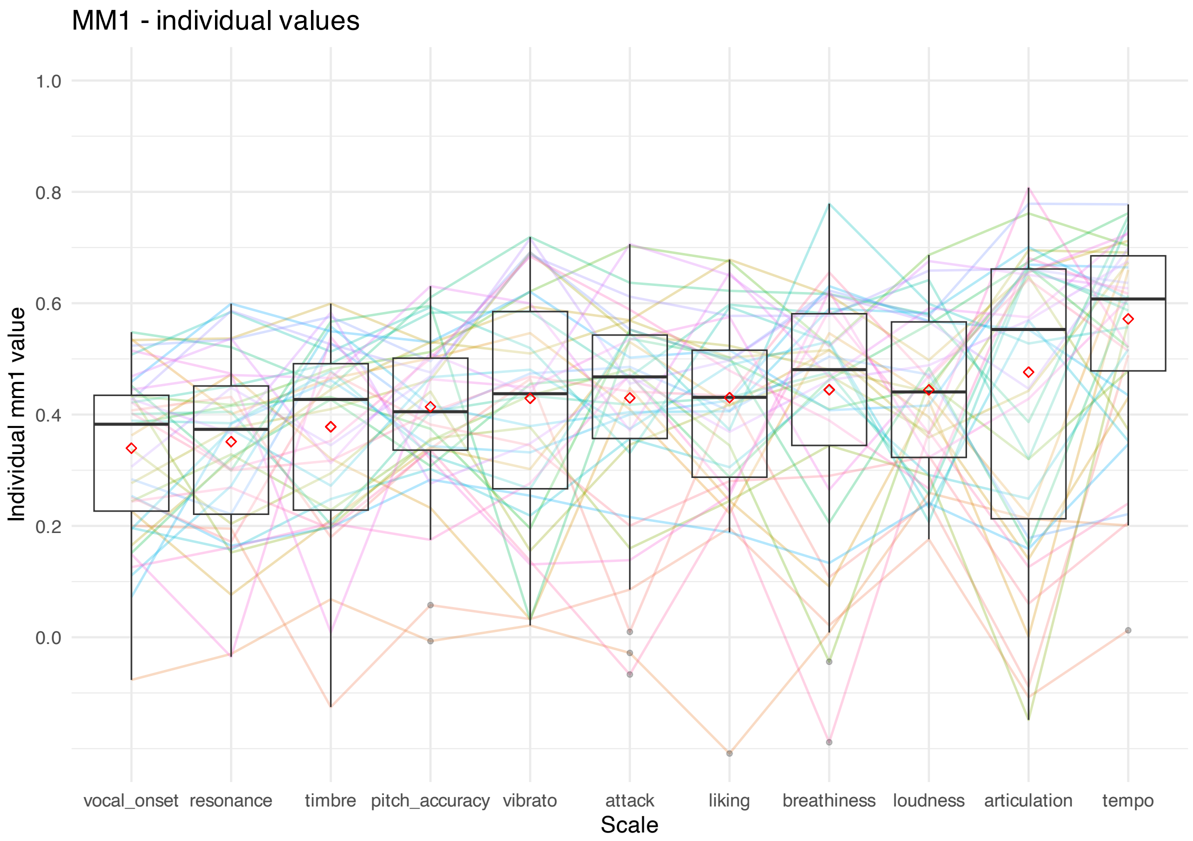

Supplementary Figure S5: Individual MM1 values for each perceptual scale and for the liking scale. The red diamonds depict the MM1 index for each scale, and each colored line represents values from an individual participant (*N* = 42). Lower and upper hinges correspond to the first and third quartiles, and whiskers extend from the hinge to 1.5 * inter-quartile range (Tukey-style boxplot).

Supplementary Table S8: Pearson correlations between acoustic measurements and mean perceptual ratings (across 42 participants) of Experiment 2 based on raw perceptual ratings and on within-participant normalized perceptual ratings. rms: root mean squared; ampl: root mean squared amplitude; CPP: cepstral peak prominence. Redundant features from different toolboxes (i.e., rms_mean from MIR Toolbox and ampl_mean from Soundgen; CPP_mean from VoiceSauce and CPP_mean from Soundgen) were included to show consistency in measurements.

**Acoustic measurement**	**Perceptual rating**	***r (raw)***	***r (norm)***
Pitch interval deviation (cents)	Pitch accuracy (out of tune - in tune)	-.20*	-.21*
Tempo (bpm)	Tempo (slow-fast)	.50***	.49***
Vibrato extent (cents)	Vibrato (not at all - a lot)	.35***	.34***
Vibrato rate (Hz)	Vibrato (not at all - a lot)	-.09	-.08
rms_mean (from MIR Toolbox)	Loudness (quiet - loud)	.24*	.25*
ampl_mean (from Soundgen)	Loudness (quiet - loud)	.25*	.27*
loudness_mean (from Soundgen)	Loudness (quiet - loud)	.41***	.43***
CPP_mean (from VoiceSauce)	Breathiness (not at all – a lot)	-.62***	-.61***
CPP_mean (from Soundgen)	Breathiness (not at all – a lot)	-.68***	-.67***
Flux_mean (from MIRToolbox)	Articulation (staccato – legato)	-.35***	-.36***
Flux_mean (from Soundgen)	Articulation (staccato – legato)	-.63***	-.63***
Spectral_flux.dmean (Essentia)	Articulation (staccato – legato)	-.43***	-.44***

*** $p < 0.001$; ** $p < 0.01$; * $p < 0.05$.

Supplementary Table S9: Summary statistics for "acoustic sensitivity": Pearson correlations between acoustic measurements and data from individual participants of Experiment 2 (based on subsets of raw perceptual ratings for each participant). rms: root mean squared; ampl: root mean squared amplitude; CPP: cepstral peak prominence.

**Acoustic measurement**	**Individual perceptual rating**	**mean r**	**sd**	**min**	**max**
Pitch interval deviation	Pitch accuracy	-0.10	0.11	-0.39	0.16
Tempo	Tempo	0.28	0.21	-0.20	0.63
Vibrato extent	Vibrato	0.14	0.18	-0.25	0.42
Vibrato rate	Vibrato	-0.03	0.09	-0.18	0.20
rms_mean (MIR Toolbox)	Loudness	0.12	0.16	-0.22	0.40
ampl_mean (Soundgen)	Loudness	0.13	0.16	-0.19	0.38
loudness_mean (Soundgen)	Loudness	0.20	0.16	-0.13	0.50
CPP_mean (VoiceSauce)	Breathiness	-0.28	0.17	-0.57	0.14
CPP_mean (Soundgen)	Breathiness	-0.31	0.21	-0.68	0.22
Flux_mean (MIRToolbox)	Articulation	-0.18	0.20	-0.50	0.27
Flux_mean (Soundgen)	Articulation	-0.31	0.23	-0.68	0.24
Spectral_flux.dmean (Essentia)	Articulation	-0.21	0.22	-0.57	0.28

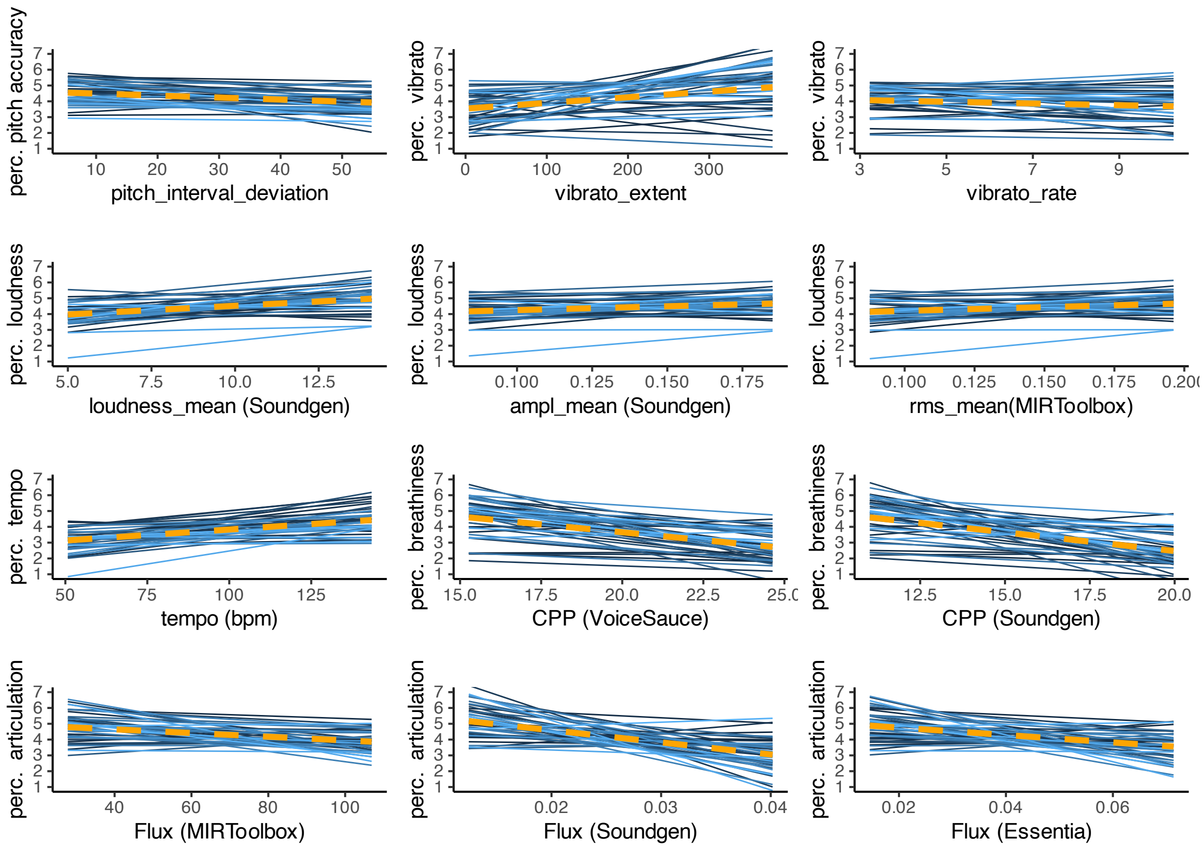

Supplementary Figure S6: "Acoustic sensitivity" or individual differences in the relationship between acoustic measurements and perceptual ratings given by participants of Experiment 2. We subset perceptual ratings by each participant and computed individual Pearson correlations with corresponding acoustic measurements. In each plot, blue lines (N = 42) represent regression lines corresponding to data from individual participants and the corresponding acoustic measurement, and the dashed orange line represents the overall correlation between the average perceptual rating of all participants and the corresponding acoustic measurement, as presented in Supplementary Table S8.

Supplementary Table S10: Coefficients of multiple linear regression model predicting the individual level of prediction obtained with the perceptual model from participants' characteristics (Experiment 2, N = 42 participants). All predictors are mean-centered and scaled by one standard deviation. 95% confidence intervals are between brackets. $F(3, 38) = 7.835$, $p < .001$.
Model syntax: lm(z_adj.r.squared_percept ~ gold_MSI + Mellow + age, data = df_part)

	**Prediction model**
(Intercept)	0.734 ***
	[0.666, 0.801]
Music sophistication	0.119 **
	[0.047, 0.192]
Mellow	-0.114 **
	[-0.187, -0.041]
Age	0.078 *
	[0.008, 0.147]
N (participants)	42
**Adj. R^2**	**0.33**

*** p < 0.001; ** p < 0.01; * p < 0.05.

Supplementary Table S11: Summary statistics for age, general music sophistication (Gold-MSI), personality traits (TIPI) and music preferences (STOMP-R) of participants of Experiment 2 (*N* = 42).

	**Mean**	**SD**	**Min**	**Max**
Age	36.8	16.1	22.0	75.0
Music Sophistication	72.9	17.6	39.0	110.0
Extraversion	4.6	1.5	1.0	7.0
Agreeableness	4.8	1.2	1.5	7.0
Conscientiousness	5.2	1.4	1.5	7.0
Emotional Stability	4.5	1.4	1.5	7.0
Openness	5.7	1.0	2.5	7.0
Mellow	4.6	1.2	1.5	6.5
Unpretensious	3.7	1.1	1.0	6.0
Sophisticated	4.6	0.8	2.6	6.0
Intense	3.6	0.7	2.0	4.8
Contemporary	4.3	1.1	1.5	6.2

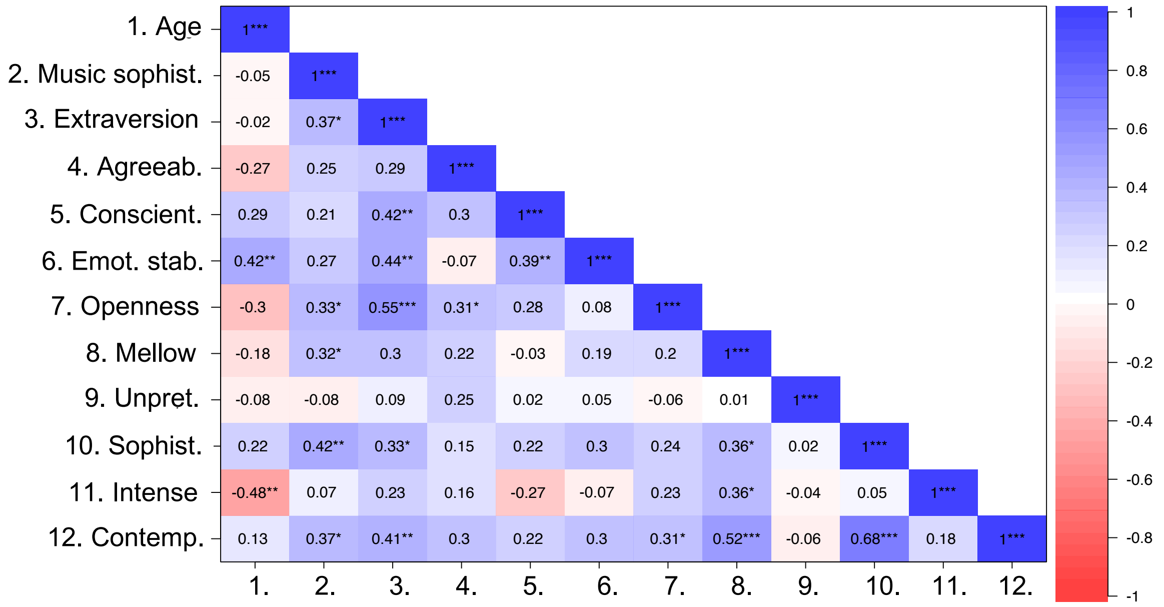

Supplementary Figure S7:: (Pearson) Correlation matrix of participant's age, general music sophistication (Gold-MSI), five personality traits (extraversion, agreeableness, conscientiousness, emotional stability, openness - TIPI) and music preferences (Mellow, Unpretensious, Sophisticated, Intense, Contemporary – MUSIC model) for participants of Experiment 2 (*N* = 42). No correction for multiple pairwise comparisons. **** p < .001; ** p < .01, * p < .05.*

There were some associations well described in the literature such as a positive correlation between trait Extraversion and a preference for Contemporary music styles ($r_{(40)}$ = .41, p < .01)(e.g.,[1]), and a negative correlation between age and preference for Intense music styles ($r_{(40)}$ = -.48, p < .01)(as in [2]). Also interesting, there was a correlation between the general music sophistication scale from Gold-MSI and preference for sophisticated music.

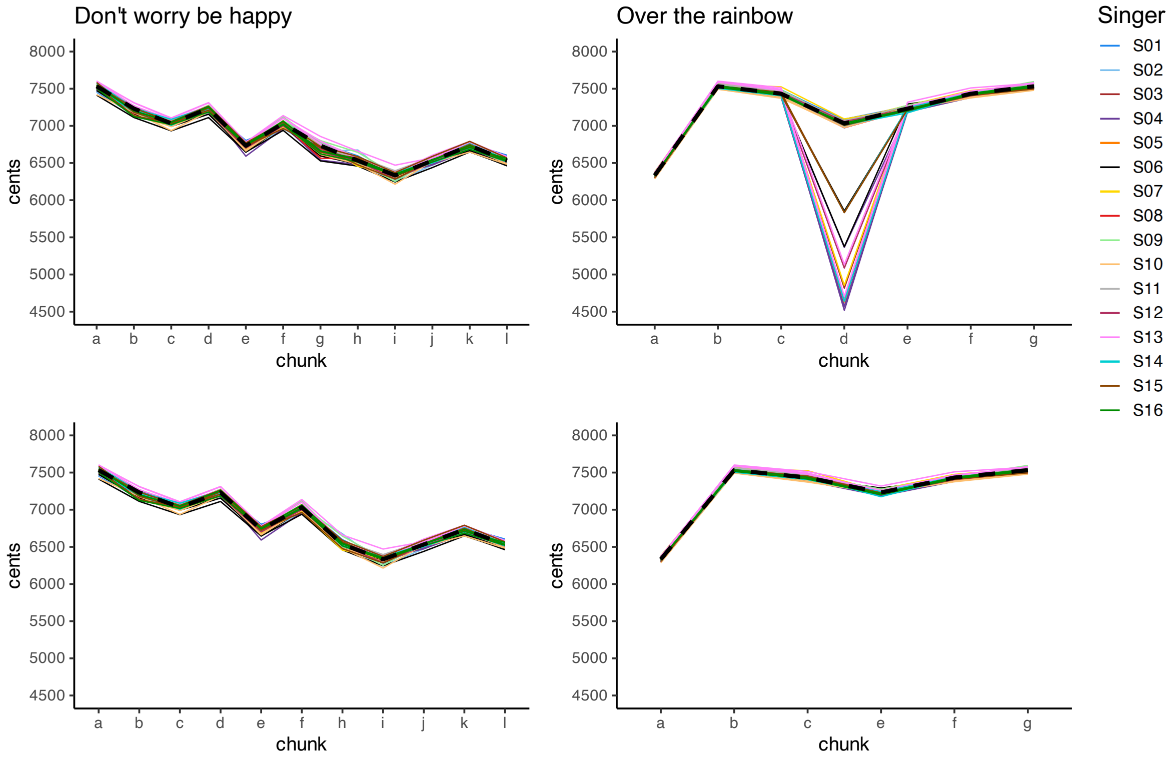

Supplementary Figure S8: Pitch interval deviation measurements for the melodies *Don't worry be happy* (left) and *Over the rainbow* (right), before(top) and after (bottom) exclusion of short notes with measurement imprecision. Each line represents one singing performance (*N* = 48 per melody, coming from 16 singers, each with three performances). Colors correspond to individual singers and the dashed black line corresponds to a reference note with perfect pitch according to sheet music. We excluded from analysis the seventh note of *Don't worry be happy* ("chunk g") and the fourth note of *Over the rainbow* ("chunk d").

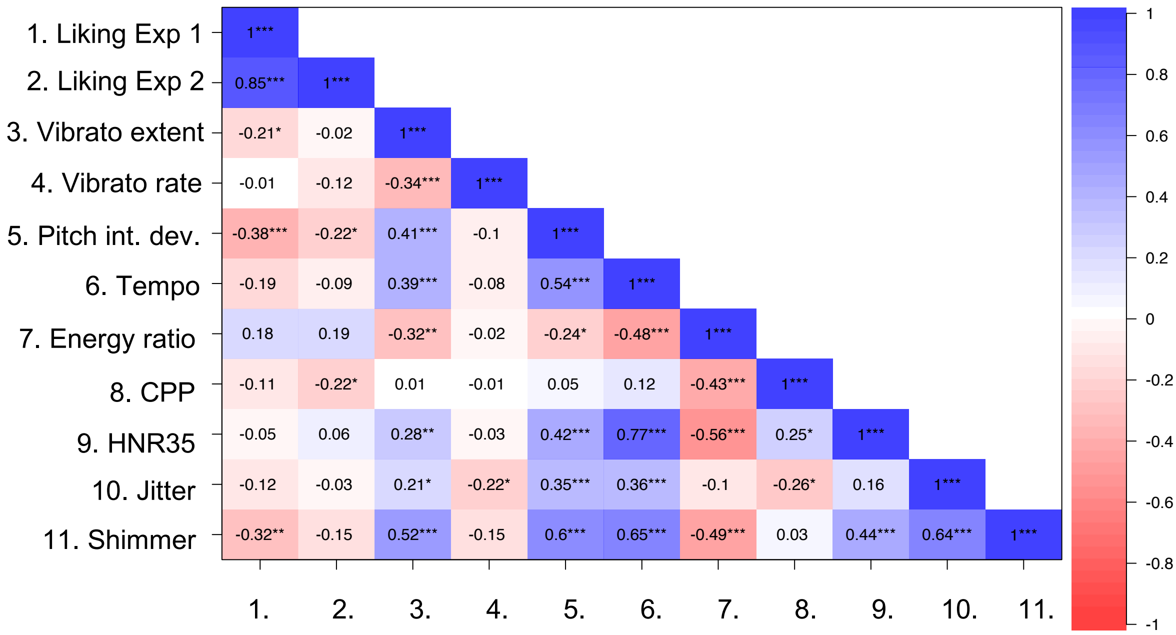

Supplementary Figure S9: (Pearson) correlation matrix of average liking ratings per stimulus item in Experiments 1 and 2 and acoustic predictors included in the acoustic model. CPP: Cepstral peak prominence, HNR35: harmonics-to-noise ratio (0 – 3.5 kHz). (**** p < .001; ** p < .01, * p < .05;* without any correction for number of comparisons).

Supplementary Methods: Selecting features from Music Information Retrieval (MIR)

Based on the 327 initially extracted MIR features from MIRToolbox [3] and Essentia [4], we first excluded clearly redundant ones, which were perfectly correlated (*r* = 1): barkbands.dmean_01-27 (correlated perfectly with frequency_bands.dmean_01-27); subband_mean/std_2-10 (correlated perfectly with mfcc_mean/std_2-10); and spectral_energyband_low.dvar (correlated perfectly with frequency_bands.dvar_01). This led to a set of 254 variables. Note these were still highly correlated with each other: among them there were 418 pairwise absolute (+ or -) correlations with *r* equal or superior to .8. We then used three alternative approaches to further reduce the remaining 254 features. All led to linear mixed models with low success in accounting for variance in liking ratings. Please see accompanying .Rmd files for all code used in feature selection ("MIR_feature_selection.Rmd" for feature reduction and "Fitting_MIR_models.Rmd" for model comparison").

A) Using the corSelect function from the fuzzySim package in R [5]

We used this function to compute pairwise correlations among the 254 MIR variables and, among each pair of variables correlated above a given threshold, exclude the variable with the least significant or least informative bivariate relationship with the response variable (supplied in the *sp.cols* argument, in our case the dependent variable, average liking ratings in Experiment 1).

Using the threshold of *r* =.5, this led to a subset of 34 MIR features:

barkbands_kurtosis.dvar, dissonance.dmean, silence_rate_20dB.dvar, silence_rate_30dB.dmean, spectral_spread.dvar, spectral_strongpeak.dmean, zerocrossingrate.dmean, frequency_bands.dmean_02, frequency_bands.dmean_05,frequency_bands.dmean_11, frequency_bands.dmean_21, frequency_bands.dmean_22, frequency_bands.dmean_27, frequency_bands.dvar_13,frequency_bands.dvar_26, frequency_bands.dvar_28, gfcc.dmean_02, gfcc.dvar_10, mfcc.dvar_13, scvalleys.dmean_02, spectral_contrast.dmean_01, low_energy_mean, brightness_mean, pitch_std,mirtempo_mean, mirtempo_std, regularity_mean, regularity_std, keyclarity_mean, kurtosis_std, mode_mean, mode_std, pulse_clarity_mean, spectral_novelty_mean

We entered this subset of features into a linear mixed model predicting (within-participant normalized) liking ratings from the selected MIR features, including random intercepts for participants and for stimuli items nested in singers; and used step-wise selection (R function step) to further reduce the model. This procedure led to the CorSelect model reported in Supplemental Tables 3 (for data of Experiment 1) and 5 (for data of Experiment 2).

B) Correlation reduction by variable elimination

We successively removed the variable (out of a given pair) with the highest Pearson pairwise correlation, until no pairwise correlations above a threshold of *r* =.5 remained.

This procedure resulted in 33 selected MIR features:

average_loudness.value, barkbands_kurtosis.dmean, barkbands_spread.dmean, dissonance.dmean, frequency_bands.dmean_05, frequency_bands.dmean_10, frequency_bands.dmean_11, frequency_bands.dmean_15, frequency_bands.dmean_28, gfcc.dvar_01, gfcc.dvar_02, gfcc.dvar_12, hfc.dmean, keyclarity_mean, mirtempo_mean, mirtempo_std, mode_mean mode_std, pitch_std, pitch.dmean, pulse_clarity_mean, regularity_mean, regularity_std, scvalleys.dmean_01, scvalleys.dmean_04, silence_rate_30dB.dmean, silence_rate_60dB.dmean, spectral_energyband_high.dmean, spectral_energyband_low.dmean, spectral_kurtosis.dmean, spectral_novelty_mean, spectral_spread.dvar, zerocross_mean

Again, we entered this subset of features into a linear mixed model predicting (within-participant normalized) liking ratings from the selected MIR features, including random intercepts for participants and stimuli items nested in singers; and used step-wise selection to further reduce the model. This procedure led to the final Corr_Reduction model reported in Supplemental Tables 3 (for data of Experiment 1) and 5 (for data of Experiment 2).

C) Correlation reduction by feature clustering

We first ran a hierarchical clustering analysis of all 254 features using Pearson correlations as the metric parameter (with the function eclust from the factoextra package [6], setting the argument hc_metric to "pearson"), which led to a solution with 10 clusters. This was followed by serial selection of features from strongly connected feature groups, by successively choosing the variable from each redundancy cluster that 1) had higher correlation to mean liking ratings and 2) was not not too highly correlated to features already selected (avoiding features where r >.7). This led to a subset of 29 features:

pitch_mean, average_loudness.value, zerocrossingrate.dmean, regularity_mean, zerocross_std, spectral_entropy.dmean, silence_rate_60dB.dmean, gfcc.dvar_02, frequency_bands.dmean_05, barkbands_spread.dmean, silence_rate_30dB.dmean, spectral_rolloff.dvar, spectral_strongpeak.dmean, zerocross_mean, dissonance.dmean, gfcc.dmean_02, hfc.dmean, frequency_bands.dvar_08, centroid_mean, frequency_bands.dmean_21, mfcc7_mean, mirtempo_std, kurtosis_std, spectral_energyband_low.dmean, skewness_mean, skewness_std, spectral_contrast.dmean_05, mfcc.dmean_05, scvalleys.dmean_02

Again, we entered this subset of features into a linear mixed model predicting (within-participant normalized) liking ratings from the selected MIR features, including random intercepts for participants and stimuli items nested in singers. We removed the variables *kurtosis_std, centroid_mean, spectral_entropy.dmean and skewness_std* because of variance inflation values above 10, and used step-wise selection to further reduce the model. This procedure led to the final MIR model H.clust reported in Supplemental Tables 2 (for data of Experiment 1) and 4 (for data of Experiment 2).

Selecting features from the Soundgen package

We repeated the steps mentioned above for the features extracted with the Soundgen R package [7]. Approaches A and B led to the same final, reduced model with only three significant predictors, amEnvDep_sd, amEnvFreq_mean and quartile25_sd. Approach C led to the same model without amEnvDep_sd as a predictor. We also built a model based on interpretability of the features, which initially included the features: amEnvFreq_mean, amEnvDep_mean, amMsFreq_mean, amMsPurity_mean, entropy_sd, fmDep_mean, fmFreq_mean, harmEnergy_sd, novelty_mean, peakFreq_sd, roughness_sd, specCentroid_mean, specSlope_sd, HNR_mean, pitch_mean.y, loudness_mean, flux_mean.y, loudness_sd, subRatio_sd, subDep_mean, harmHeight_sd, CPP_mean, entropySh_mean, quartile50_sd, fmDep_mean, quartile25_sd, novelty_sd, harmEnergy_mean. We entered this subset of features into a linear mixed model predicting (within-participant normalized) liking ratings from these selected features, including random intercepts for participants and for stimuli items nested in singers; and used step-wise selection (R function step) to further reduce the model. This procedure led to the Soundgen model reported in Supplemental Tables 3 (for data of Experiment 1) and 5 (for data of Experiment 2). Note this model is similar to the resulting model from approaches A and B, with the exception that it includes amEnvDep_mean instead of amEnvDep_sd (these two are highly correlated); and also included specCentroid_mean and entropySh_mean.

Supplementary Table S12 : Comparison of prediction achieved and variance components of models based on raw and within-participant normalized ratings. All models follow the same syntax reported in Supplementary Tables S2 (or S4), S3 (or S5) and S7. Marg: marginal; cond: conditional; ICC: Intraclass Correlation Coefficient (indicating proportion of variance captured by random intercepts). Note that in the normalized version of perceptual model in Experiment 2, all ratings were normalized (i.e., liking as well as perceptual ratings).

	**Normalized ratings**					**Raw ratings**				
**Model**	**Marg R^2**	**Cond R^2**	**ICC (part)**	**ICC (Singer /item)**	**ICC (Singer)**	**Marg R^2**	**Cond R^2**	**ICC (part)**	**ICC (Singer /item)**	**ICC (Singer)**
Acoustic mod. Exp 1	.016	.238	.127	.036	.063	.011	.444	362	.029	.047
Acoustic mod. Exp 1 consist.	.025	.301	.098	.070	.114	.020	.436	.282	.057	.086
Acoustic mod. Exp 2	.032	.304	.129	.072	.081	.026	.403	.259	.057	.071
Perceptual mod. Exp 2	.433	.587	.257	.025	.015	.445	.631	.314	.020	.013

Supplementary Table S13: Perceptual features collected in Experiment 2 (German version)

Rating	Definition	Anchor words
Tonhöhengenauigkeit	Wie präzis ist jeder Ton entlang der Melodie? Ist die Aufführung gut gestimmt?	schlecht gestimmt – gut gestimmt
Behauchte Stimmlage	Die Menge des Luftstroms in der Stimme. Wie gehaucht klingt die Stimme?	gar nicht gehaucht - sehr gehaucht
Artikulation	Wie folgende Töne miteinander verbunden sind. Sind Töne getrennt (staccato) oder verbunden (legato)?	staccato – legato
Spracheinsatz I	Der Beginn eines Tones. Ist der Anfang der Töne weich oder hart?	weich - hart
Spracheinsatz II	Der Beginn eines Tones. Ist der Anfang der Töne genau/präzis?	unpräzis - präzis
Lautstärke -	Die Intensität des Hörempfindens. Ist die Stimme leise oder laut?	leise - laut
Tempo	Die Geschwindigkeit der Aufführung. Ist die Aufführung langsam oder schnell?	langsam - schnell
Klangfülle	Die Fülle oder der Resonanz einer Stimme. Wie klangvoll ist die Stimme?	klangarm - klangvoll
Klangfarbe	Die wahrgenommene Klangqualität der Stimme. Klingt die Stimme dunkel oder hell?	hell - dunkel
Vibrato	Eine leichte und periodische Schwingung der Tonhöhe eines gehaltenen Tones. Wie viel Vibrato verwendet die Sängerin?	gar kein – sehr viel

SI References

1. Greenberg, D. M. *et al.* Universals and variations in musical preferences: A study of preferential reactions to Western music in 53 countries. *Journal of Personality and Social Psychology* **122**, 286–309 (2022).
2. Bonneville-Roussy, A., Rentfrow, P. J., Xu, M. K. & Potter, J. Music through the ages: Trends in musical engagement and preferences from adolescence through middle adulthood. *Journal of Personality and Social Psychology* **105**, 703–717 (2013).
3. Lartillot, O., Toiviainen, P. & Eerola, T. A Matlab toolbox for Music Information Retrieval. in *Data Analysis, Machine Learning and Applications* (eds. Preisach, C., Burkhardt, H., Schmidt-Thieme, L. & Decker, R.) 261–268 (Springer Berlin Heidelberg, Berlin, Heidelberg, 2008). doi:10.1007/978-3-540-78246-9_31.
4. Bogdanov, D. *et al.* Essentia: an audio analysis library for music information retrieval. in *Proceedings of the 14th International Society for Music Information Retrieval Conference* 493–498 (2013).
5. Barbosa, A. M. fuzzySim: applying fuzzy logic to binary similarity indices in ecology. *Methods Ecol Evol* **6**, 853–858 (2015).
6. Kassambara, A. & Mundt, F. Factoextra: Extract and visualize the results of multivariate data analyses. (2020).
7. Anikin, A. Soundgen: An open-source tool for synthesizing nonverbal vocalizations. *Behav Res* **51**, 778–792 (2019).
